# Novel Polymorphic Cocrystals of the Non-Steroidal Anti-Inflammatory Drug Niflumic Acid: Expanding the Pharmaceutical Landscape

**DOI:** 10.3390/pharmaceutics13122140

**Published:** 2021-12-13

**Authors:** Francisco Javier Acebedo-Martínez, Carolina Alarcón-Payer, Antonio Frontera, Rafael Barbas, Rafel Prohens, Milena Di Crisci, Alicia Domínguez-Martín, Jaime Gómez-Morales, Duane Choquesillo-Lazarte

**Affiliations:** 1 Laboratorio de Estudios Cristalográficos, IACT, CSIC-Universidad de Granada, Avda. de las Palmeras 4, 18100 Armilla, Spain; j.acebedo@csic.es (F.J.A.-M.); milena.dicrisci@student.unisi.it (M.D.C.); jaime@lec.csic.es (J.G.-M.); 2 Servicio de Farmacia, Hospital Universitario Virgen de las Nieves, 18014 Granada, Spain; carolina.alarconpayer@gmail.com; 3 Department of Chemistry, Universitat de les Illes Balears, Crta de Valldemossa km 7.5, 07122 Palma de Mallorca, Spain; toni.frontera@uib.es; 4 Unitat de Polimorfisme i Calorimetria, Centres Científics i Tecnològics, Universitat de Barcelona, Baldiri Reixac 10, 08028 Barcelona, Spain; rafa@ccit.ub.edu; 5 Department of Inorganic Chemistry, Faculty of Pharmacy, University of Granada, 18071 Granada, Spain

**Keywords:** cocrystal polymorphism, niflumic acid, caffeine, NSAIDs, mechanochemical synthesis

## Abstract

Any time the pharmaceutical industry develops a new drug, potential polymorphic events must be thoroughly described, because in a crystalline pharmaceutical solid, different arrangements of the same active pharmaceutical ingredient can yield to very different physicochemical properties that might be crucial for its efficacy, such as dissolution, solubility, or stability. Polymorphism in cocrystal formulation cannot be neglected, either. In this work, two different cocrystal polymorphs of the non-steroidal anti-inflammatory drug niflumic acid and caffeine are reported. They have been synthesized by mechanochemical methods and thoroughly characterized in solid-state by powder and single crystal X-ray diffraction respectively, as well as other techniques such as thermal analyses, infrared spectroscopy and computational methods. Both theoretical and experimental results are in agreement, confirming a conformational polymorphism. The polymorph NIF–CAF Form I exhibits improved solubility and dissolution rate compared to NIF–CAF Form II, although Form II is significantly more stable than Form I. The conditions needed to obtain these polymorphs and their transition have been carefully characterized, revealing an intricate system.

## 1. Introduction

**Scheme 1:**
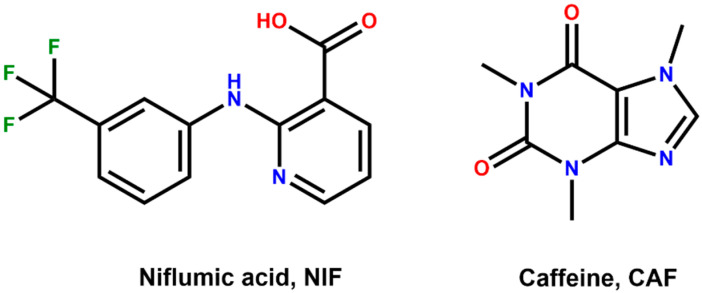
Chemical formula of niflumic acid (NIF) and caffeine (CAF).

Niflumic acid (NIF, 2-{[3-(trifluoromethyl)phenyl]amino}-3-pyridinecarboxylic acid, Scheme 1) is a widely prescribed non-steroidal anti-inflammatory drug (NSAID). Its mechanism of action is linked to the non-competitive and reversible inhibition of the ciclooxygenase-2 enzyme. It is mainly used in clinics to relieve the pain and inflammation associated with rheumatoid arthritis and other related acute or progressive inflammatory processes such as gouty arthritis or osteoarthritis [1]. According to the Biopharmaceutics Classification System (BCS), NIF is a class II drug, defined by low solubility and high permeability values [2], which directly affect the poor oral bioavailability of this drug and thus, its therapeutic efficacy.

The development of multicomponent pharmaceutical solids is a novel strategy addressed by the pharmaceutical industry to enhance drug performance [3]. This is achieved through crystal engineering of the intimate structure of pharmaceutical solids on which the physicochemical properties rely, without modifying the chemical identity of those active pharmaceutical ingredients (APIs) involved in the formulation [4]. Among all kinds of multicomponent pharmaceutical solids, cocrystals have emerged as a promising approach to efficiently modulate the pharmaceutical properties of APIs already existing in the market at a relatively low cost, such as solubility, dissolution profile, and stability [5,6].

Polymorphism studies also are of paramount importance in the pharmaceutical industry since different polymorphs of the same API, with distinct crystal structures, can yield to completely different physicochemical properties [7,8]. At first, cocrystals were thought to be a solution to polymorphism problems presented by APIs. It was assumed that the incorporation of coformers could stabilize the API in a particular crystalline structure due to the establishment of non-covalent intermolecular interactions, thus preventing the transformation of one polymorph into another. However, this premise was revealed to be false [9], and the number of polymorphic cocrystals being reported has significantly increased in the past few years [10,11,12], Therefore, polymorphism in pharmaceutical solids must be considered at all levels [13,14].

Caffeine (CAF, 1,3,7-Trimethylpurine-2,6-dione, Scheme 1) is a natural xanthine derivative commonly present in food and pharmaceutical formulations as a psychostimulant ingredient. CAF has an outstanding safety profile; thus, it has been widely used as a coformer in cocrystallization studies [15,16]. The successful isolation of novel cocrystals of niflumic acid with caffeine has already been reported in a Russian patent, which claims an enhancement of the solubility properties of the novel pharmaceutical solid compared to the parent API [17]. There also exist relevant examples of NIF cocrystals with other coformers with the same purpose [18,19,20,21]. Nevertheless, it is well-known that the fenamate family of drugs, to which NIF belongs, exhibits polymorphism [22,23]; hence, it is of interest to study whether polymorphism would also affect the cocrystals derived from this API or not, in order to estimate the potential of this formulation in the pharmaceutical industry.

In the present work, two novel crystalline polymorphs of the cocrystal NIF–CAF are reported. The single crystal structure of both polymorphs is thoroughly described, thus providing useful insights about the structural differences that drive their physicochemical properties, mainly stability, solubility, and dissolution rate. Special attention has been paid to the experimental conditions that yield to the isolation of the different polymorphs as well as their thermodynamic relationships. In addition, the impact of intermolecular interactions and their energetic contribution have been calculated by theoretical methods, supporting the structural analysis.

## 2. Materials and Methods

### 2.1. Materials

Niflumic acid, caffeine, and solvents used are commercially available from Sigma-Aldrich. All solvents were used as received without additional purification.

### 2.2. General Procedure for Synthesis of Cocrystal Polymorphs

Mechanochemical syntheses were conducted by liquid-assisted grinding (LAG) in a Retsch MM200 ball mill operating at 25 Hz frequency using stainless steel jars along with two stainless steel balls of 7 mm diameter. All syntheses were repeated to ensure reproducibility. For liquid-assisted grinding screening, a selection of solvents (Table S1) with different polarities were used.

Synthesis of NIF–CAF Form I: A mixture of NIF (141.11 mg, 0.50 mmol) and CAF (97.10 mg, 0.50 mmol) in a 1:1 stoichiometric ratio was placed in a 10 mL stainless steel jar along with 150 μL of dichloromethane and two stainless steel balls of 7 mm diameter. The mixture was then milled for 30 min. Form I was also obtained by annealing a ground solid mixture of NIF and CAF in an oven at 130 °C for 24 h.

Synthesis of NIF–CAF Form II: A mixture of NIF (141.11 mg, 0.50 mmol) and CAF (97.10 mg, 0.50 mmol) in a 1:1 stoichiometric ratio was placed in a 10 mL stainless steel jar along with 150 μL of acetonitrile and two stainless steel balls of 7 mm diameter. The mixture was then milled for 30 min.

Co-grinding of the blends of NIF–CAF in dichloromethane and in acetonitrile was monitored. Different samples of the mixture of the components were milled separately for different time periods up to 30 min. Samples were then analyzed by PXRD to examine changes in crystallinity and evaluate cocrystal formation. The raw data obtained in all these samples were analyzed through the Rietveld method in order to quantify the phases present at each time period.

### 2.3. Powder X-ray Diffraction (PXRD)

Powder X-ray diffraction data were collected using a Bruker D8 Advance Vαrio diffractometer (Bruker-AXS, Karlsruhe, Germany) equipped with a LYNXEYE detector and Cu-Kα1 radiation (1.5406 Å). All the profile fittings were conducted using the software Diffrac.TOPAS 6.0 [24]. The bulk phase purity was checked by Rietveld refinement, using cell parameters from structural crystallographic information of the constitutive phases, namely NIF and CAF, as well as the new reported phases. In these fittings, only the background, unit cell parameters, and zero error were refined. Rwp values obtained in all cases demonstrate an excellent agreement between the structural model and the bulk phase measured by powder diffraction.

Thermodiffractometric data for NIF–CAF Form II were obtained on a sample loaded on an Anton Paar HTK1200N Camera, under inert atmosphere, on a PANalytical X’Pert Pro automated diffractometer with CuKα1 and the X’Celerator detector. Data were collected from 112 to 178 °C with a heating rate of 2 °C·min^−1^ and a delay time of 5 min to ensure thermal stabilization.

### 2.4. Preparation of Single Crystals

Single crystals were grown by fast and slow solvent evaporation at room temperature using the polycrystalline material obtained from mechanical synthesis. Suitable crystals for X-ray diffraction studies were obtained from recrystallization in saturated solutions (Table S2). Fast solvent evaporation, when the vials containing the solution were left uncovered, afforded NIF–CAF Form I in approximately one day; meanwhile, slow solvent evaporation, using vials sealed with a perforated Parafilm^TM^, resulted in NIF–CAF Form II after 5 days in acetonitrile.

### 2.5. Single-Crystal X-ray Diffraction (SCXRD)

Measured crystals were prepared under inert conditions immersed in perfluoropolyether as protecting oil for manipulation. Suitable crystals were mounted on MiTeGen Micromounts™, and these samples were used for data collection. Data for NIF–CAF Form I and NIF–CAF Form II were collected with a Bruker D8 Venture diffractometer with graphite monochromated CuKα radiation (λ = 1.54178 Å). The data were processed with APEX3 suite [25]. The structures were solved by intrinsic phasing using the ShelXT program [26], which revealed the position of all non-hydrogen atoms. These atoms were refined on F^2^ by a full-matrix least-squares procedure using anisotropic displacement parameter [27]. All hydrogen atoms were located in difference Fourier maps and included as fixed contributions riding on attached atoms with isotropic thermal displacement parameters 1.2 or 1.5 times those of the respective atom. Both polymorphs exhibit positional disorder over the –CF_3_ group, which was modelled as two alternative positions (0.65:0.35 ratio, for NIF–CAF Form I and 0.75:0.25 ratio, for NIF–CAF Form II). The OLEX2 software was used as a graphical interface [28]. Intermolecular interactions were calculated using PLATON [29]. Molecular graphics were generated using Mercury [30]. The crystallographic data for the reported structures were deposited with the Cambridge Crystallographic Data Center as supplementary publication no. CCDC 2116472 and 2116473. Additional crystal data are shown in Table 1. Copies of the data can be obtained free of charge at http://www.ccdc.cam.ac.uk/products/csd/request.

### 2.6. Computational Studies

The calculations of the non-covalent interactions were carried out using the Gaussian-16 [31] and the PBE0-D3/def2-TZVP level of theory [32,33]. To evaluate the interactions in the solid state, the crystallographic coordinates were used. The interaction energies were computed by calculating the difference between the energies of isolated monomers and their assembly. The interaction energies were calculated with correction for the basis set superposition error (BSSE) by using the Boys–Bernardi counterpoise technique [34]. Bader’s “Atoms in molecules” theory (QTAIM) [35] was used to study the interactions discussed herein by means of the AIMall calculation package [36]. The molecular electrostatic potential surfaces were computed using the Gaussian-16 software [31].

In order to assess the nature of interactions in terms of being attractive or repulsive and reveal them in real space, we used the NCIplot index, which is a method for plotting non-covalent interaction regions [37], based on the NCI (Non-Covalent Interactions) visualization index derived from the electronic density [38]. The reduced density gradient (RDG), coming from the density and its first derivative, is plotted as a function of the density (mapped as isosurfaces) over the molecule of interest. The sign of the second Hessian eigenvalue times the electron density (i.e., sign(λ_2_)ρ in atomic units) enables the identification of attractive/stabilizing (blue-green-colored isosurfaces) or repulsive (yellow-red-colored isosurfaces) interactions using 2D plots.

### 2.7. FT-IR Analysis

Fourier transform infrared (FTIR) spectroscopic measurements were performed on a Bruker Tensor 27 FTIR instrument (Bruker Corporation, Billerica, MA, USA) equipped with a single-reflection diamond crystal platinum ATR unit and OPUS data collection program. The scanning range was from 4000 to 400 cm^−1^ with a resolution of 4 cm^−1^.

### 2.8. Thermal Analysis

Differential scanning calorimetry (DSC) analysis was carried out by means of a Mettler-Toledo DSC-822e calorimeter (Mettler Toledo, Columbus, OH, USA). Experimental conditions: aluminum crucibles of 40 μL volume, atmosphere of dry nitrogen with 50 mL/min flow rate, heating rates of 1 °C/min and 10 °C/min. The calorimeter was calibrated with indium of 99.99% purity (m.p.: 156.4 °C; ΔH: 28.14 J/g).

Thermogravimetric Analysis (TGA) was performed on a Mettler-Toledo TGA-851e thermobalance (Mettler Toledo, Columbus, OH, USA). Experimental conditions: alumina crucibles of 70 μL volume, atmosphere of dry nitrogen with 50 mL/min flow rate, heating rates of 1 °C/min and 10 °C/min.

Hot-stage microscopy (HSM) was performed on a Nikon polarization microscope (Nikon Eclipse 50i, Tokyo, Japan) equipped with a Linkam LTS350 hot stage (Linkam Scientific Instruments Ltd., Tadworth, U.K.), and a digital video recorder was used. Experimental conditions: atmosphere of dry nitrogen and heating rates of 1 °C/min and 10 °C/min.

### 2.9. Stability Test

Slurry experiments were conducted using excess powder samples of mixture of cocrystal polymorphs in a ratio 1:1 in 1 mL of solvents (Table S3) for 24 h at room temperature in a sealed vial containing a magnetic stirrer. The solids in the vials were collected, filtered, and dried at 35 °C for subsequent analysis by PXRD.

Stability of cocrystal polymorphs was also studied at accelerated storage conditions; 200 mg of each solid was taken in watch glasses and the physical stability was evaluated at 40 °C in 75% relative humidity using a Memmert HPP110 climate chamber (Memmert, Schwabach, Germany). The samples were subjected to the above accelerated stability conditions for 6 months. PXRD was used to monitor the stability of the solid forms.

### 2.10. Powder Dissolution Profile

The powder dissolution profiles of NIF and the cocrystal polymorphs in water PBS pH 7.4 were collected using milled and sieved (75–150 μm) powders. During the powder dissolution experiments, an excess amount of solid sample was suspended in 10 mL of water in a flask and stirred at 600 rpm using an overhead stirrer at 25 °C. An aliquot of the slurry was withdrawn at predetermined time points (0.5, 1, 2, 3, 4, 5, 6, 7, 8, 12, and 24 h) and immediately passed through a 0.22 μm PES (polyether sulfone) filter membrane. The filtrates were diluted, and the absorbance was measured using a Varian Cary 50 ultraviolet (UV) visible spectrophotometer (Agilent Technologies, Santa Clara, CA, USA). The calibration curve was prepared using standard solutions of NIF, which were analyzed at 267 nm to avoid coformer interference. The absorbance measurements of the diluted solutions from the saturated ones were used to quantify the amounts of solubilized samples, considering the dilution factor. After the powder dissolution experiments, the undissolved powders were recovered and analyzed by PXRD.

## 3. Results and Discussion

### 3.1. Preparation of Cocrystal Polymorphs

**Figure 1:**
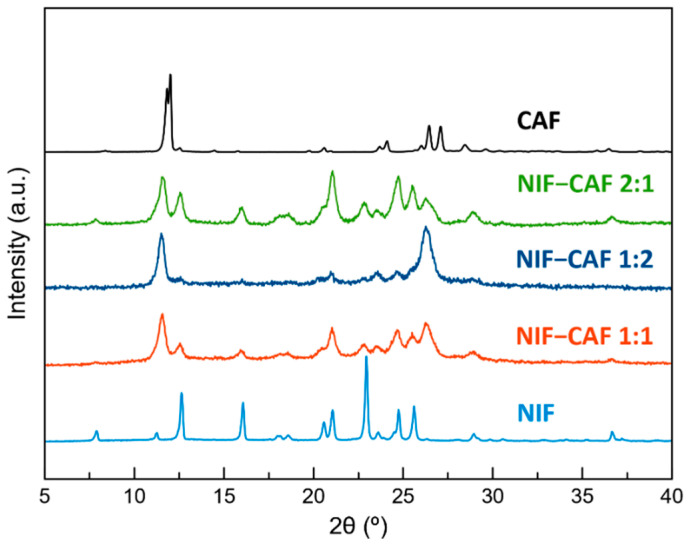
Powder X-ray diffraction (PXRD) patterns of the NIF and CAF physical mixtures obtained by neat grinding at different molar ratios, and the corresponding reactants.

Mechanochemistry has proved to be a powerful tool to prepare multicomponent solid materials, including salts, cocrystals, and hydrates/solvates, as well as their respective combinations. It has also shown to be of interest for the obtention of polymorphs, particularly dealing with the discovery of new solid drug dosage forms [39]. Cocrystallization of NIF with CAF was carried out by neat and LAG using different stoichiometries (1:1, 1:2 and 2:1). Neat grinding resulted in a low crystallinity physical mixture of the APIs (Figure 1), while the solvent screening grinding (Table S1) resulted in two new NIF–CAF cocrystal polymorphs.

**Figure 2:**
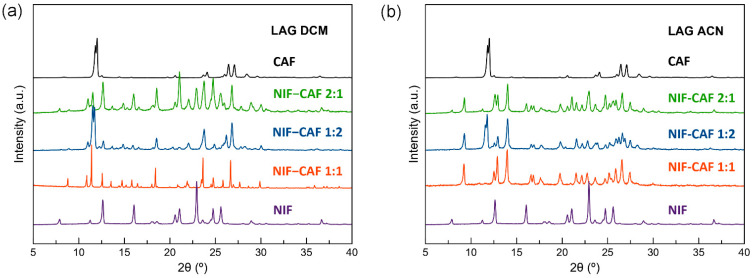
PXRD patterns of the two new solid forms obtained by liquid-assisted grinding (LAG) the two components at different molar ratios and in (**a**) dichloromethane (DCM) and (**b**) acetonitrile (ACN).

The patterns of NIF–CAF obtained by LAG at different molar ratios of the two components in a selection of solvents were compared with the patterns of isolated NIF and CAF. The comparison shows that all three ratios have common characteristic peaks that were different from the two parent APIs. The 1:2 and 2:1 NIF–CAF patterns also contained peak characteristics to the 1:1 phase. Only the 1:1 products had a completely different pattern, and it was exactly the same as that simulated from the crystal structures of the new multicomponent polymorphs reported in this work (Figure 2). The analysis by PXRD of the samples generated during LAG experiments of 1:1 blends revealed two group of solvents that afforded the different phases reported in this work (Table S1). Meanwhile, H_2_O resulted in a mixture of NIF and caffeine monohydrate (Figure S1).

**Figure 3:**
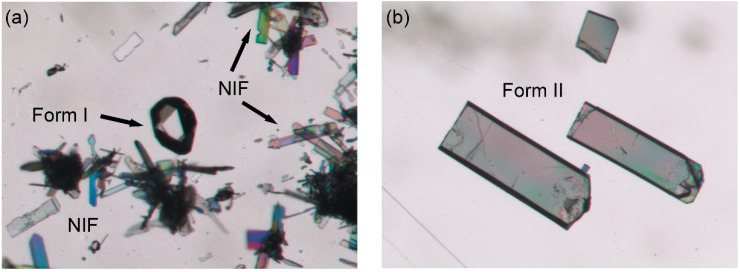
Optical photomicrograph of crystalline material obtained by (**a**) fast evaporation (acetone) and (**b**) slow evaporation (acetonitrile).

The new phases were used for further recrystallization to obtain suitable crystals for structural determination. Again, a selection of solvents was used (Table S2). Fast solvent evaporation afforded concomitant crystallization of NIF and as further proved by SCXRD, a NIF–CAF cocrystal polymorph, hereafter referred to as Form I (Figure 3a). On the other hand, slow solvent evaporation resulted in the crystallization of only one phase, a second polymorph of NIF–CAF, hereafter referred to as Form II (Figure 3b). Comparison of calculated patterns from crystal structures with those from the LAG solids showed that Form I and Form II were the phases obtained during grinding experiments (Figure S2). Annealing solid Form II at 130 °C for 24 h (Figure S3) proved to be an additional procedure to obtain Form I.

**Figure 4:**
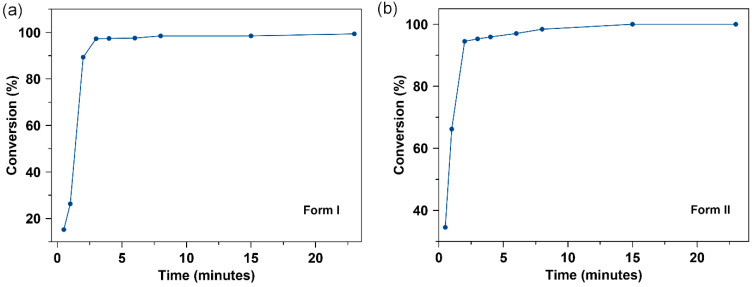
NIF–CAF cocrystal polymorphs formation during co-grinding at room temperature. (**a**) Form I (LAG in dichloromethane) and (**b**) Form II (LAG in acetonitrile).

The extent of cocrystallization of NIF–CAF polymorphs during co-grinding is shown in Figure 4. Quantification was undertaken by Rietveld analysis on the obtained X-ray diffraction pattern as described in the experimental section. These results indicate the quick formation of both forms with a faster rate of cocrystallization in the early stages of the process, and approximately 99% cocrystal formed after co-grinding for 15 min.

### 3.2. Crystal Structure Analysis

**Figure 5:**
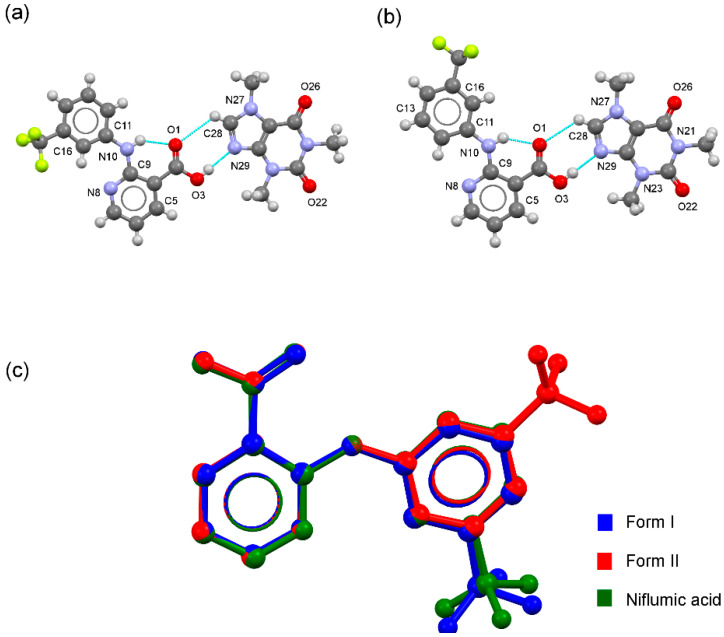
(**a**) Asymmetric unit of the Form I cocrystal. (**b**) Asymmetric unit of the Form II cocrystal. (**c**) Molecular overlay of the cocrystal polymorphs and the reported structure of niflumic acid.

The two polymorphs of NIF–CAF crystallize in the monoclinic space group *P2_1_/n* with 1:1 stoichiometry (Figure 5). The carboxylic acid homosynthon of NIF is disrupted in preference to the formation of the supramolecular heterosynthon between the carboxyl moiety of NIF and the imidazole ring of CAF. Interestingly, this heterosynthon is present in each form, with only a slight variation in hydrogen bond distances (Table S4). The O3–H3···N29 and the weak C28–H28···O1 interactions align API and coformer, forming a supramolecular dimer based on the $D_{2}^{2}\left(7\right)$ graph set motif [40,41]. The analysis of the C–O bond distances of the carboxylic acid group of NIF supports the cocrystal formation [42]. In the NIF–CAF system, C–O distances were indicative of a protonated acid, as expected for a cocrystal with ΔD_C–O_ values of 0.11 Å and 0.09 Å for Form I and Form II, respectively, in contrast to the ΔD_C–O_ values observed in salts (typically less than 0.03 Å).

Intramolecular N–H···O=C and C–H···N_pyridine_ hydrogen bonds keep the anthranilic acid fragment planar in the NIF molecules of the two cocrystal polymorphs. The main difference between them lies in the conformation of the NIF molecule. The rotation of the other aromatic ring around the N10–C11 bond gives rise to two conformers. The overlay of the NIF molecules in both polymorphs reveals that the two aromatic rings show a significant orientational difference in conformation due to the free rotation on the N–C bond (Figure 5c). Considering the torsion angle τ = C9–N10–C11–C16 as a reference, Form I displays the six-membered aromatic rings substituents (–CF_3_ and carboxylic acid) on opposite sides (τ = −173.51°), as observed in the reported structure of niflumic acid [43,44], whereas Form II locates them on the same side (τ = 8.26°). Thus, taking into account these conformational differences, NIF–CAF forms could be classified as conformational polymorphs. Nevertheless, this is not the only polymorphic class that could be used to describe the NIF–CAF system.

**Figure 6:**
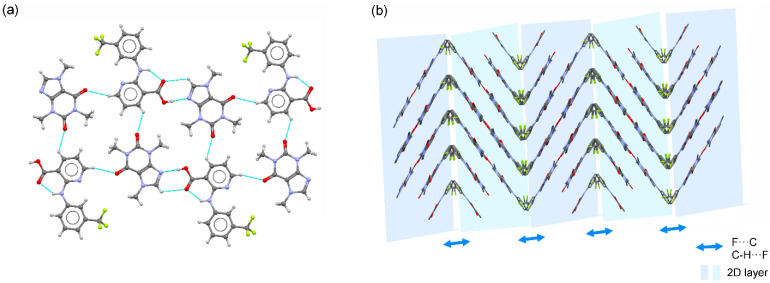
(**a**) Tape structure built by H-bonding interactions in Form I. (**b**) Detail of the 2D layer structure in Form I. H atoms omitted for clarity.

In Form I, the dimeric unit is further connected with two adjacent dimeric units through C–H···O hydrogen bonds involving the *para*-hydrogen atom of the anthranilic acid ring (C7–H7···O26). Pairs of chains are associated through additional C5–H5···O22 hydrogen bonds to form a tape structure that locate the –CF_3_ groups pointing into the tape, stabilizing the structure by C–H···F contacts. Tetrameric assemblies build by stacking interactions of type NIF···CAF···CAF··NIF connect the tape structures to form a 2D layer structure. Finally, F···C contacts and C–H···F as well as C-H···π interactions bind these layers to create the 3D structure (Figure 6).

**Figure 7:**
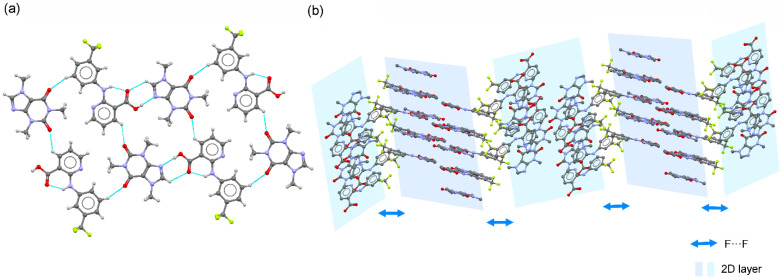
(**a**) Tape structure built by H-bonding interactions in Form II. (**b**) Detail of the 2D layer structure in Form II. H atoms omitted for clarity.

As expected, the different conformation of the NIF molecule in Form II affects its crystal structure. In Form II, the dimeric unit is also connected with two adjacent dimeric units through C13–H13···O26 hydrogen bonding interactions involving the –CF_3_ ring of NIF and the carbonyl group of CAF to form a chain. As in the case of Form I, pairs of chains are associated through additional C5–H5···O22 hydrogen bonds to form a tape structure, locating this time the –CF_3_ groups in the periphery. Multi-π,π stacking interactions involving the anthranilic acid fragment of NIF and both rings of CAF connect the tapes to generate a 2D layer structure. This structure is also pilled by contribution of F···F contacts, thus forming the tridimensional structure shown in Figure 7. Comparing the differences in the arrangement of the infinite NIF–CAF layers, it is also possible to classify the polymorphs of the NIF–CAF cocrystal as packing polymorphism.

### 3.3. DFT Study of Noncovalent Interactions

**Figure 8:**
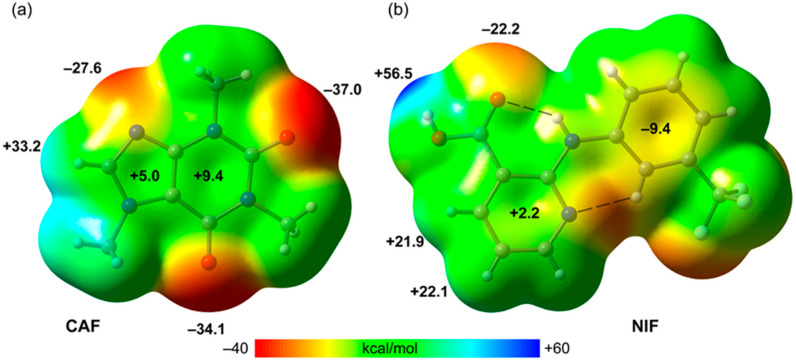
MEP surfaces (isovalue: 0.01 a.u.) of CAF (**a**) and NIF (**b**) at the PBE0-D3/def2-TZVP level of theory. The energies at selected points of the surfaces are given in kcal/mol.

First, we have computed the MEP surface of both co-formers in order to investigate the most nucleophilic and electrophilic parts of the molecule. Moreover, we are also interested in the electronic rich/poor nature of the aromatic rings. Figure 8 shows the MEP surfaces of both co-formers, evidencing that the MEP maximum is located at the carboxylic H-atom of the NIF molecule. Interestingly, the MEP is also large and positive at the CH bond of the imidazole ring of CAF. The MEP minimum is located at the O-atom bonded to C2 of CAF (−37.0 kcal/mol). The MEP value is also large and negative at the N9-atom of the imidazole ring (−27.6 kcal/mol). The MEP value is smaller (in absolute value) at the O-atom of the carboxylic group of NIF, likely due to its participation in intramolecular H-bonding. In case of CAF, the MEP values are modest and positive over the six- and five-membered rings of CAF. In the NIF coformer, the value is positive over the carboxypyridine ring and negative over the phenyl ring. In addition, the MEP values at the aromatic H-atoms of the carboxypyridine ring are also significantly positive (~22 kcal/mol). The enhanced acidity of these protons and the π-acidic nature of this ring is likely due to the effect of the intramolecular H-bonds, inducing a charge transfer from the carboxypyridine ring to the trifluoromethylphenyl ring.

**Figure 9:**
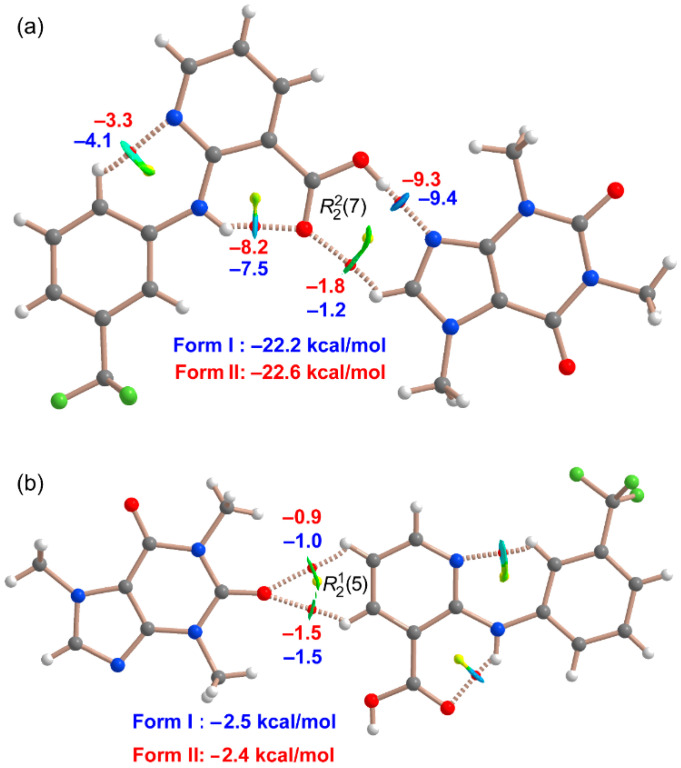
QTAIM distribution of bond and ring critical points (CPs, red and yellow spheres, respectively) corresponding to the H-bonds in $R_{2}^{2}$(7) (**a**) and $R_{1}^{2}$ (5) synthons (**b**). The dissociation energies of the H-bonds are indicated next to the bond CPs. Superimposed NCIplot isosurfaces [s = 0.5, cut-off = 0.04 a.u., color scale −0.04 a.u. (blue) ≤ (signλ2) ρ ≤ 0.04 a.u.(red)] are also indicated.

A common feature in the solid state of both compounds is the formation of $R_{2}^{2}$(7) and $R_{1}^{2}$(5) H-bonded synthons, as represented in Figure 9, along with the distribution of bond and ring critical points (red and yellow spheres, respectively) and bond paths. Moreover, the superimposed noncovalent interaction plot (NCIplot) index isosurfaces are also represented. The NCIplot index method is very convenient since it characterizes the noncovalent interactions in real space and reveals their attractive or repulsive nature by using a color scale. The QTAIM analysis of the H-bonded dimer shown in Figure 9a confirms the existence of two inter and two intramolecular bonds. The intermolecular O-H···N and C-H···O H-bonds generate the $R_{2}^{2}$(7) synthon where the carboxylate group of NIF interacts with the complementary N9 and CH groups of the imidazole ring of CAF. Each H-bond is characterized by a bond CP and bond path connecting the H-atom to the N,O-atoms. Moreover, they are also characterized by small NCIplot isosurfaces that are coincident with the location of the bond CPs. Regarding the $R_{2}^{2}$(7) synthon, the isosurface color is dark blue for the OH···N contact and green for the CH···O contact, revealing strong and weak interactions, respectively, in line with the MEP values shown in Figure 8. The intramolecular H-bonds are responsible for the coplanarity of the aromatic rings in NIF. The formation energy of each individual H-bond was computed using the potential energy density values at the bond CPs (Vr) and the equation proposed by Espinosa et al. (E = 0.5 × Vr). We have indicated the energies next to the bond CPs in Figure 9, using blue for Form I and red for Form II. The formation energies of the $R_{2}^{2}$(7) synthon are similar for both polymorphs (Form II 0.5 kcal/mol more stable). Regarding the intramolecular H-bonds, the NH···O is stronger in Form II, which is compensated by the CH···N H-bond that is stronger in Form I. Considering all H-bonds, Form II is 0.4 kcal/mol more stable. Regarding the $R_{1}^{2}$(5) dimer, the most nucleophilic O-atom of CAF establishes a bifurcated H-bond with the aromatic H-atoms belonging to the carboxypyridine ring, in line with the MEP surface analysis. In this case, the energy associated to this $R_{1}^{2}$(5) synthon is smaller than that of the $R_{2}^{2}$(7) synthon, as expected. The energy difference between both polymorphs is very small (0.1 kcal/mol). Therefore, it can be concluded that the H-bonding network in both polymorphs is energetically equivalent.

**Figure 10:**
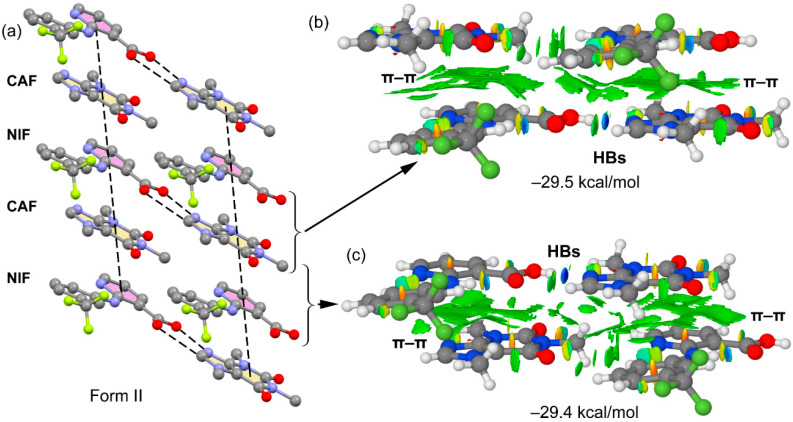
(**a**) Partial view of the X-ray packing of Form II. (**b**,**c**) NCIplot isosurfaces [s = 0.5, cut-off = 0.04 a.u., color scale −0.04 a.u. (blue) ≤ (signλ2) ρ ≤ 0.04 a.u. (red)] of the tetrameric assemblies extracted from the X-ray packing.

**Figure 11:**
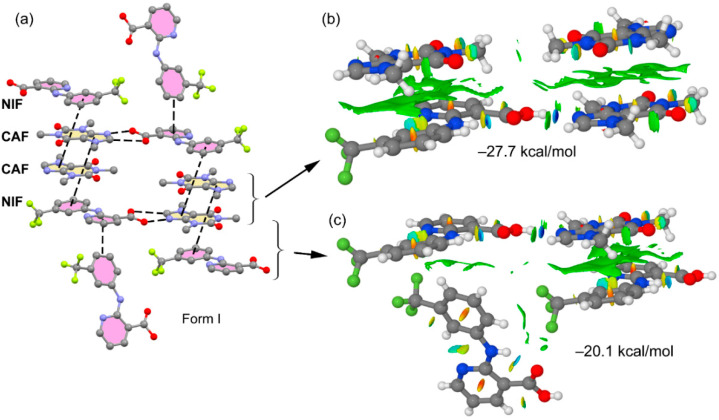
(**a**) Partial view of the X-ray packing of Form I. (**b**,**c**) NCIplot isosurfaces [s = 0.5, cut-off = 0.04 a.u., color scale −0.04 a.u. (blue) ≤ (signλ2) ρ ≤ 0.04 a.u. (red)] of the tetrameric assemblies extracted from the X-ray packing.

The main difference between both polymorphs resides in the π-stacking assemblies that they form in the solid state, as detailed in Figure 10 and Figure 11. Form II (Figure 10a) forms infinite columns where the $R_{2}^{2}$(7) H-bonded dimer interacts with both CAF and NIF molecules above and below the supramolecular plane in such a way that CAF···(NIF···CAF)_n_···NIF assemblies are generated. The NCIplot index analyses of two tetrameric assemblies are shown in Figure 10, evidencing large green isosurfaces that are typical of π-stacking interactions. The formation energy of the tetramers (considering the $R_{2}^{2}$(7) H-bonded dimer as previously formed) is basically identical for both tetramers (−29.5 kcal/mol and −29.4 kcal/mol), thus confirming the relevance of such assemblies.

Figure 11a shows the X-ray packing of Form I, where the $R_{2}^{2}$(7) H-bonded dimer interacts with two CAF molecules at one side of the supramolecular plane and two NIF molecules at the opposite side, forming discrete NIF···CAF···CAF···NIF assemblies. We have computed the binding energies of the tetrameric assemblies shown in Figure 11b,c and characterized them using the NCIplot index. It can be observed that the binding energies of these assemblies are significantly smaller (in absolute value) than those of Form II. Therefore, in terms of π-stacking interactions, Form II is significantly more stable than Form I. This result is in agreement with the experimental results observed in the stability study for both polymorphs (see Section 3.6).

### 3.4. FT-IR Analysis

FT–IR technique can be used to monitor the formation of multicomponent pharmaceutical solids by identifying changes in those vibrational frequencies belonging to IR bands with high diagnostic value within APIs and coformers. These changes are essentially attributed to the presence of non-covalent interactions in the crystal structure, i.e., the formation of supramolecular synthons [45,46].

In the IR spectrum of NIF, the IR bands corresponding to the carboxylic group have important diagnostic value, involving the stretching C=O at 1665 cm^−1^, the stretching of C-O at 1244 cm^−1^, and the in-plane and out-of-plane bending of the O-H at 1448 and 935 cm^−1^, respectively. It was also possible to determine both the stretching and the bending mode of N-H at 3324 and 1529 cm^−1^, respectively. Conversely, the C-F bands were hidden in the fingerprint region from 1400 to 1000 cm^−1^. Regarding the IR spectrum of CAF, we could observe the υ(C=O) mode as two close bands at 1701 and 1655 cm^−1^, and different bands were attributed to the methyl groups: υ_as_(CH_3_) at 2957 cm^−1^, δ_as_(CH_3_) at 1481 cm^−1^ and δ_s_(CH_3_) at 1361 cm^-−1^. In addition, the stretching mode of aromatic C-H was also found in CAF at 3118 cm^−1^. Interestingly, the FT–IR spectra of Form I and Form II cocrystals of NIF–CAF are rather similar between them but show some shifts in comparison with the spectra of isolated NIF and CAF. A comparison of the FT–IR vibrational frequencies among the parent components and the novel polymorphic cocrystals is given in Table 2, while the FT–IR spectra are shown in Figure S6.

In both NIF–CAF cocrystals, the ν(NH) mode of NIF is overshadowed and could not be determined. The region at about 3000 cm^−1^ of both polymorphs resembles that of caffein, where the bending modes of aromatic C-H and asymmetric bending of the methyl groups are identified. Below 1700 cm^−1^, spectra are crowded with rather intense bands. Because of their diagnostic value, it should be highlighted that these bands relate to the bending of the N-H group and the different bands linked to the carboxylic group (Table 2), which demonstrate the presence of these dissociable protons also in the NIF–CAF polymorphs. This evidence agrees with the reported crystal structures in which we can observe both groups actively involved in non-covalent intermolecular interactions.

### 3.5. Thermal Analysis

**Figure 12:**
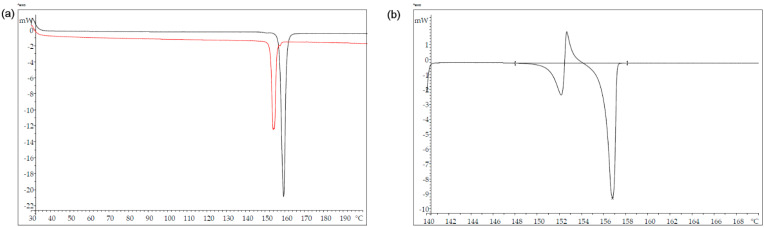
(**a**) DSC thermograms of Form I (black) and Form II (red) at a 10 °C/min heating rate. (**b**) DSC thermogram of Form II at a 1 °C/min heating rate.

Differential scanning calorimetry was used to study the relative stability of both forms. When heated at 10 °C/min, both forms show a single endothermic phenomenon, which corresponds to melting, at 152 °C for Form II and at 157 °C for Form I (Figure 12a). However, when Form II is heated at 1 °C/min, a melting process followed by recrystallization of Form I and its subsequent melting is observed (Figure 12b).

The higher enthalpy of fusion of the lower melting form revealed an enantiotropic relationship between both polymorphs based on Burger and Ramberger’s heat of fusion rule [47]. Thus, in order to estimate the transition temperature between the enantiotropic pair using the theoretical approach by Yu et al. [48], it is necessary to determine the temperatures and enthalpies of fusion of both polymorphs (Equation (1)). The parameter *k* was fixed to 0.003, since it has been experimentally reported to provide a good approximation of the heat capacity differences in the majority of cases [49]. The theoretical value obtained by following this calculation was 119 °C. Table 3 shows the melting point and enthalpy values used.

(1) $$T_{trs}=\frac{\Delta H_{fus,2}-\Delta H_{fus,1}+k\Delta H_{fus,1}\left(T_{fus,1}-T_{fus,2}\right)}{\frac{\Delta H_{fus,2}}{T_{fus,2}}-\frac{\Delta H_{fus,1}}{T_{fus,1}}+k\Delta H_{fus,1}\ln\left(\frac{T_{fus,1}}{T_{fus,2}}\right)}$$

We also experimentally determined the transition temperature by slurrying equimolar mixtures of both forms in octane at different temperatures and measuring the resulting solid by PXRD. The transformation from Form II to Form I was observed to be completed in 24 h at 90 °C, which is well aligned with the theoretical value of 119 °C (Figure S7).

**Figure 13:**
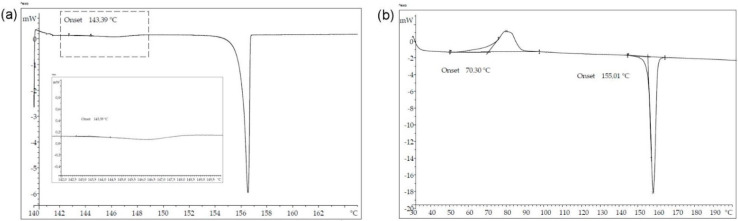
(**a**) DSC thermogram of Form II at a 0.2 °C/min heating rate. (**b**) DSC thermogram of the solid obtained by quenching from the melt of Form I.

Moreover, a very low heating rate (0.2 °C/min) DSC experiment was conducted with Form II and an endothermic phenomenon around 143 °C was observed, followed by the melting of Form I, which is an additional proof of the enantiotropic nature of the polymorphic relationship (Figure 13a). Densities calculated from the crystal structures are also aligned with these experimental findings since the stable form at low temperature (Form II) is the one with the higher density.

We also characterized both polymorphs by thermogravimetric analysis to discard decomposition after melting of Form I and thus to explore the metastable solid form landscape obtained after quenching from the melt. TGA curves show a weight loss right after the melting of Form I but not simultaneous with it (Figures S9 and S11). This encouraged us to melt Form I in the DSC crucible under nitrogen flow and immediately quench it in a liquid nitrogen bath. Then, we immediately ran a DSC experiment at 10 °C/min (Figure 13b), which showed the recrystallization at 70 °C of Form I but without further evidence of other metastable forms.

**Figure 14:**
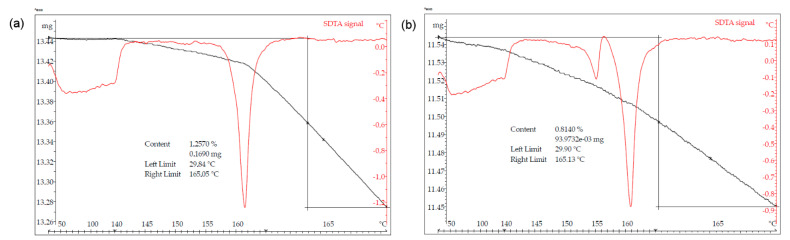
(**a**) Variable heating rate TGA thermograms of Form I (**a**) and Form II (**b**). Method: first step from 30 °C to 140 °C at 10 °C/min heating rate, a second step from 140 °C to 165 °C at 1 °C/min heating rate and finally a third isothermic step at 165 °C for 20 min. SDTA signal is represented in red.

Interestingly, variable heating rate TGA analysis revealed from 30 °C to 165 °C a small weight loss of 1.3% in Form I (Figure 14a) and 0.8% in Form II (Figure 14b), suggesting a potential sublimation of both polymorphs, since sublimation of pure caffeine had been previously described [50], and destabilization of the caffeine/malonic acid cocrystal via sublimation had been also reported by Alsirawan et al. [51]. Thus, in order to gain a deeper insight into the thermal behavior of the polymorphic system, we conducted variable temperature powder X-ray diffraction in combination with optical thermomicroscopy experiments.

**Figure 15:**
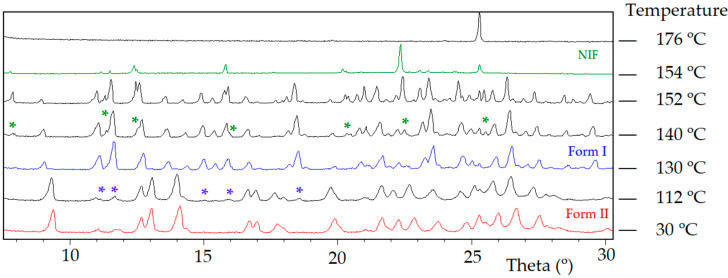
Variable temperature PXRD experiment starting from Form II. Peaks of each new phase are highlighted in blue asterisk (Form I) and green asterisk (niflumic acid). Diffraction peak observed at 176 °C is caused by the sample holder. Enlargement is 8–30° 2θ.

First, starting with Form II, we monitored its evolution by measuring PXRD diagrams at different temperature values and observed that it started to transform into Form I at around 112 °C and totally transformed at 130 °C, while a new crystal phase was detected at 140 °C, which, according to the simulated PXRD diagram from the crystal structure, corresponds to the niflumic acid. However, diffraction peaks of pure caffeine were not observed during the whole process. At higher temperature values, a decrease in the intensity of diffraction peaks of Form I together with an increase in those for niflumic acid was observed. Finally, at around 176 °C, diffraction peaks are not observable anymore, revealing the melt of the remaining niflumic acid (Figure 15).

**Figure 16:**
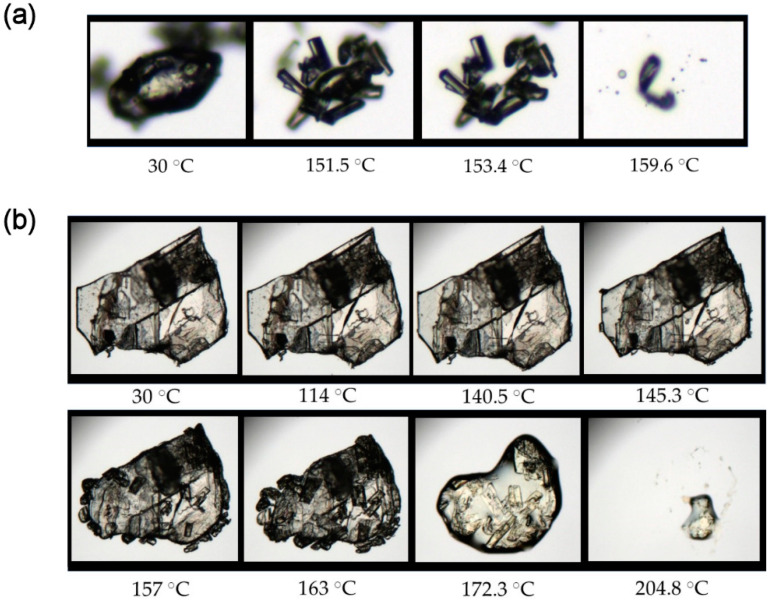
Microphotographs of the evolution of crystals of Form II (**a**) and Form I (**b**) when heated in on a hot-stage instrument.

The presence of niflumic acid diffraction peaks but not signals of caffeine in combination with the weight loss measured by TGA and the bibliographic antecedents of caffeine cocrystals destabilization via sublimation strongly supported the hypothesis of dissociation of the niflumic acid/caffeine cocrystal into crystalline niflumic acid and sublimated caffeine. This was confirmed through thermomicroscopy experiments conducted on a hot-stage instrument. Figure 16a shows the most relevant captured microphotographs when Form II was subjected to a 1 °C/min heating program under nitrogen flow. This experiment essentially reveals the melting of Form II followed by recrystallization of Form I and its final melting, in total accordance with the DSC experiments.

However, when Form I was subjected to the same heating ramp (Figure 16b), the growth of new crystals from the surface of the existing ones is observed at 140 °C, followed by the melting starting at 157 °C of the original crystals, leaving the new ones perfectly intact until they start to melt at 172 °C (niflumic acid melting point is 204 °C [52]).

**Figure 17:**
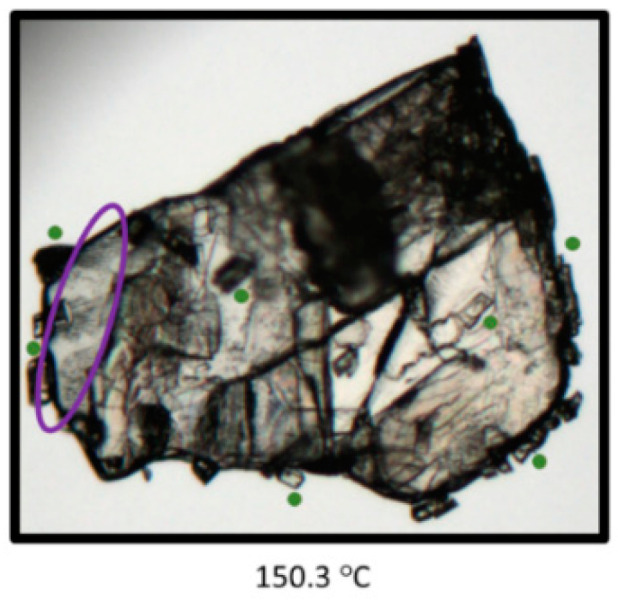
Microphotograph of a crystal of Form I when heated in on a hot-stage instrument at 150.3 °C. Sublimation area is highlighted in purple and new crystals of niflumic acid are located at the green points.

This thermomicroscopic behavior visually describes what was observed by variable temperature PXRD experiments and it is in agreement with the DSC/TGA experiments, suggesting that Form I (but not Form II, or at least to a lesser extent) is destabilized via sublimation of caffeine and recrystallization of niflumic acid (Figure 17).

**Figure 18:**
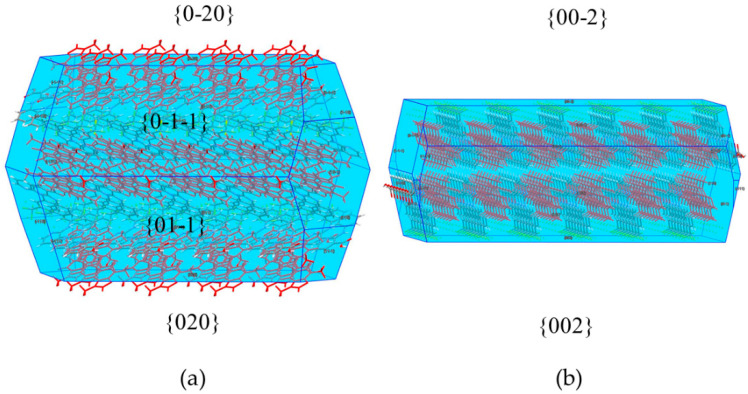
BFDH-predicted morphologies of Form I (**a**) and Form II (**b**), showing the largest faces. Caffeine molecules are represented in red and niflumic acid molecules in green. The {011} and {0-11} faces of Form I are omitted for clarity.

BFDH morphologies of each polymorph were calculated by using the Bravais–Friedel–Donnay–Harker (BFDH) method included in the latest release of the visualization software package Mercury [53] in order to detect significant differences that could explain the different behavior regarding the recrystallization of niflumic acid on the surface of the cocrystal. In the case of Form I (Figure 18a), the predicted morphology shows only caffeine molecules pointing out of the {020} and {0-20} faces (corresponding to 31% of the total surface), and both caffeine and niflumic acid molecules at the {011}, {01-1}, {0-1-1}, and {0-11} faces (corresponding to 34% of the total surface). On the other hand, in Form II, only niflumic acid molecules are located at all the faces of the predicted morphology (Figure 18b). Thus, the total absence of caffeine molecules at the predicted faces of Form II as opposed to Form I can have an impact on the surface properties of each polymorph, preventing caffeine sublimation in Form II, which could provide a plausible explanation of the observed differences on the nucleation and growth of niflumic acid crystals.

### 3.6. Stability Studies

The stability of cocrystal polymorphs was studied in this work by performing slurry experiments at 25 °C and storing them at accelerated ageing conditions (40 °C and 75% relative humidity).

Competitive slurry experiments using an equimolar mixture of both polymorphs in a selection of solvents showed that after 3 days of agitation at 25 °C, NIF–CAF Form II was afforded (Figure S12). These results are in agreement with the thermal analysis. These polymorphs have an enantiotropic relationship, with NIF–CAF Form II being the stable polymorph at room temperature and Form I at high temperatures.

In the accelerated stability tests, the powder samples of the cocrystals were stored at 40 °C and 75% relative humidity (RH). Results of the stability tests suggest that NIF–CAF polymorphs remained the same after storage for 6 months (Figure S13).

### 3.7. Powder Dissolution

**Figure 19:**
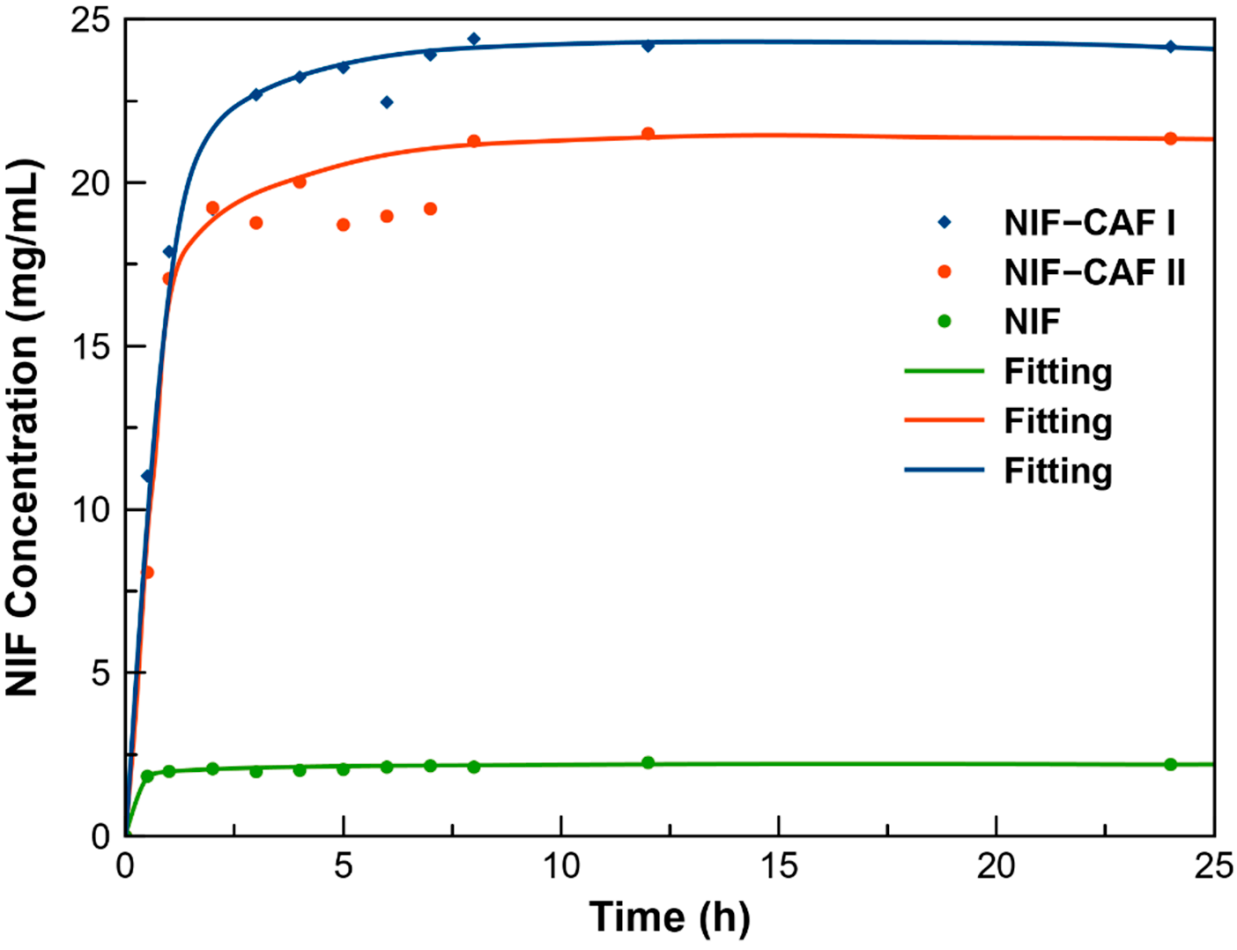
Powder dissolution profile of NIF–CAF cocrystal polymorphs and NIF, and the corresponding fitting curves (solid lines).

Powder dissolution experiments were conducted to evaluate the solubility and dissolution behavior of the two cocrystal polymorphs at pH 7.4 phosphate buffer medium under constant temperature and stirring rate. Powder dissolution profiles of NIF, NIF–CAF Form I, and NIF–CAF Form II are shown in Figure 19 as NIF concentrations (mg/mL) against time (h). According to Kitamura et al. [54,55], the crystals are expected to be completely dispersed since a high stirring rate was applied (600 rpm), thus establishing a true equilibrium. As it can be observed, NIF–CAF Form I and NIF–CAF Form II can achieve a higher NIF concentration with a faster rate. Their dissolution plots reached the maximum apparent solubility (S_max_) within 8 h. The maximum apparent solubility (S_max_) of NIF and the two cocrystal polymorphs obtained are summarized in Table S6. We can see that the S_max_ values of NIF–CAF Form I and NIF–CAF Form II are 10.84 and 9.56 times higher than the parent NIF. As a less stable polymorph, NIF–CAF Form I exhibits better apparent solubility and a higher dissolution rate as compared to NIF–CAF Form II. The supersaturated solutions were formed at the beginning of the dissolution process, and then the concentrations of the polymorphs keep almost constant up to the end of the experiment at 24 h (Figure 19). The PXRD analysis of the remaining powder after the dissolution experiments confirms the partially slow phase conversion of the cocrystals to pure NIF (Figure S14). Interestingly, the higher S_max_ combined with the absence of a “spring and parachute effect” in the dissolution profile opens the door to the use of these polymorphs as potential sustained release formulations [56] while reducing the dosage of the parent API in the formulation.

## 4. Conclusions

The study of potential polymorphism in the development of new pharmaceutical solids is of paramount importance for the pharmaceutical industry, including the multicomponent pharmaceutical solids. The API of reference in this study, niflumic acid, belongs to the fenamate family of drugs, which are well known to show conformation polymorphism. Therefore, in the development of NIF cocrystals, the study of possible polymorphic cocrystals seems to be mandatory. Indeed, two different polymorphs of NIF–CAF cocrystals were isolated and thoroughly described, with theoretical and experimental results being in good agreement.

NIF–CAF Form I and Form II polymorphs differ in the conformation of the NIF molecule, thus affecting the arrangement of the NIF–CAF layers in the corresponding crystal structures and subsequently having an impact on the surface properties of each polymorph. In fact, the transition between polymorphs seems to be related to the caffeine sublimation in the formulation. As it should be expected from a metastable phase within an enantiotropic system, polymorph NIF–CAF Form I shows better solubility and dissolution profile than Form II. Nonetheless, Form II is the most stable polymorph at room temperature. Interestingly, both polymorphs were stable for 6 months under accelerating ageing conditions. This fact, along with the improved solubility and the lack of dose dumping effects, would allow not only a reduced amount of the parent API in drug formulations, but also make these NIF–CAF polymorphic cocrystals interesting candidates for potential novel-sustained, or even prolonged, release formulations. Despite these promising results, further analyses need to be carried out to confirm the potential interest of NIF–CAF cocrystals for the pharmaceutical industry.

## Figures and Tables

**Table 1:** Crystallographic data and structure refinement details of NIF–CAF cocrystal polymorphs.

Compound Name	NIF–CAF Form I	NIF–CAF Form II
Formula	C_21_H_19_F_3_N_6_O_4_	C_21_H_19_F_3_N_6_O_4_
Formula weight	476.42	476.42
Crystal system	Monoclinic	Monoclinic
Space group	P2_1_/n	P2_1_/n
a/Å	8.618(3)	6.9667(4)
b/Å	23.908(9)	8.1222(6)
c/Å	10.872(4)	37.438(3)
α/°	90	90
β/°	104.568(12)	94.400(4)
γ/°	90	90
V/Å^3^	2168.1(14)	2112.2(2)
Z	4	4
D_c_/g cm^−3^	1.460	1.498
µ/mm^−1^	1.042	1.070
F(000)	984	984
Reflections collected	15737	19086
Unique reflections	3763	3710
R_int_	0.0796	0.0435
Data/restraints/parameters	3763/93/340	3710/81/340
Goodness-of-fit (F^2^)	0.974	1.111
R1 (I > 2σ(I))	0.0637	0.0467
*w*R2 (I > 2σ(I))	0.1633	0.1282
CCDC	2116472	2116473

**Table 2:** Summary of relevant FT-IR vibrational frequencies (cm^−1^) in the spectra of NIF, CAF, and NIF–CAF Forms I and II.

Compound	ν(C-H) Aromatic	ν_as_(CH_3_)	ν(C=O) Carboxyl	ν(C=O) Keto	δ(N-H)	δ(O-H) In-Plane	ν(C-O) Carboxyl	δ(O-H) Out-Of-Plane
NIF			1665		1529	1448	1241	935
CAF	3118	2957		1701 1655				
NIF–CAF Form I	3134	2955	1662	1710	1530	1461	1244	947
NIF–CAF Form II	3131	2955	1662	1709	1533	1446	1242	947

**Table 3:** Parameters used to assess the relative stability of both polymorphs.

Polymorph	Melting Point (K)	Enthalpy of Fusion (J/g)
I	430.83	145.70
II	425.32	161.84

## Data Availability

Not applicable.

## Supplementary Materials

The following are available online at https://www.mdpi.com/article/10.3390/pharmaceutics13122140/s1, Figure S1: PXRD pattern of the solid material obtained by liquid-assisted grinding (LAG) with water, the simulated patterns from crystal structures and the corresponding reactants. Figure S2: Rietveld profile fit (red line) to the experimental PXRD data (blue line) of NIF–CAF Form I (a) and NIF–CAF Form II (b). The profile fitting for both the cocrystals shows low discrepancy (grey line). Figure S3: PXRD patterns of NIF–CAF after annealing of Form II at 130 ºC during 24 h. Figure S4: ORTEP representation showing the asymmetric unit of NIF–CAF Form I with atom numbering scheme (thermal ellipsoids are plotted with the 50% probability level). Figure S5: ORTEP representation showing the asymmetric unit of NIF–CAF Form II with atom numbering scheme (thermal ellipsoids are plotted with the 50% probability level). Figure S6: Comparison of Fourier transform infrared (FT−IR) spectra of NIF, CAF and NIF–CAF polymorphs. Figure S7: PXRD patterns of the solid material obtained after slurry assay in octane (OCT) at selected temperature. Figure S8: DSC of NIF–CAF Cocrystal Form I. Figure S9: TGA of NIF–CAF Cocrystal Form I. Figure S10: DSC of NIF–CAF Cocrystal Form II. Figure S11: TGA of NIF–CAF Cocrystal Form II. Figure S12: PXRD patterns of NIF–CAF cocrystal forms after the competitive slurry experiments using an equimolar mixture of both polymorphs in a selection of solvents. Figure S13: PXRD patterns of NIF–CAF cocrystal polymorphs with respect to the stability under accelerated ageing conditions (40 °C, 75% RH) at different time intervals. Figure S14: PXRD patterns of NIF–CAF cocrystal polymorphs after the powder dissolution profile as-say. Table S1: Solvents added in the LAG syntheses and the resulting polymorph of the NIF-CAF cocrystal solvent screening grinding. Table S2: Solvents used for single crystal growth by solvent evaporation method. Table S3: Solvents used for the competitive slurry experiments using an equimolar mixture of both polymorphs. Table S4: Hydrogen bonds for NIF–CAF cocrystal polymorphs [Å and deg.]. Table S5: π,π-stacking interactions analysis of compound NIF-CAF Form II. Table S6: Maximum apparent solubility (Smax) of NIF and its NIF–CAF Cocrystal polymorphs in pH 7.4 phosphate buffer medium.
