# Supplementary material for: Novel Polymorphic Cocrystals of the Non-Steroidal Anti-Inflammatory Drug Niflumic Acid: Expanding the Pharmaceutical Landscape
Source: Pharmaceutics. 2021 Dec 13;13(12):2140. doi: 10.3390/pharmaceutics13122140 (PMC8706418; doi:10.3390/pharmaceutics13122140)
Supplement: Supplementary file 1 [file pharmaceutics-13-02140-s001.zip › pharmaceutics-1490236-SI.pdf]

pharmaceutics

MDPI

Article

Supplementary Materials: Novel Polymorphic cocrystals of the non-steroidal anti-inflammatory Drug Niflumic acid: expanding pharmaceutical landscape

Francisco Javier Acebedo-Martínez, Carolina Alarcón-Payer, Antonio Frontera, Rafael Barbas, Rafel Prohens, Milena Di Crisci, Alicia Domínguez-Martín, Jaime Gómez-Morales and Duane Choquesillo-Lazarte

Figure S1. PXRD pattern of the solid material obtained by liquid assisted grinding (LAG) with water, the simulated patterns from crystal structures and the corresponding reactants.

Figure S2. Rietveld profile fit (red line) to the experimental PXRD data (blue line) of NIF–CAF Form I (a) and NIF–CAF Form II (b). The profile fitting for both the cocrystals shows low discrepancy (grey line).

Figure S3. PXRD patterns of NIF–CAF after annealing of Form II at 130 ºC during 24 h.

Figure S4. ORTEP representation showing the asymmetric unit of NIF–CAF Form I with atom numbering scheme (thermal ellipsoids are plotted with the 50% probability level).

Figure S5. ORTEP representation showing the asymmetric unit of NIF–CAF Form II with atom numbering scheme (thermal ellipsoids are plotted with the 50% probability level).

Figure S6. Comparison of Fourier transform infrared (FT−IR) spectra of NIF, CAF and NIF–CAF polymorphs.

Figure S7. PXRD patterns of the solid material obtained after slurry assay in octane (OCT) at selected temperature.

Figure S8. DSC of NIF–CAF Cocrystal Form I.

Figure S9 TGA of NIF–CAF Cocrystal Form I.

Figure S10. DSC of NIF–CAF Cocrystal Form II.

Figure S11. TGA of NIF–CAF Cocrystal Form II.

Figure S12. PXRD patterns of NIF–CAF cocrystal forms after the competitive slurry experiments using an equimolar mixture of both polymorphs in a selection of solvents.

Figure S13. PXRD patterns of NIF–CAF cocrystal polymorphs with respect to the stability under accelerated ageing conditions (40 °C, 75% RH) at different time intervals.

Figure S14. PXRD patterns of NIF–CAF cocrystal polymorphs after the powder dissolution profile assay.

Table S1. Solvents added in the LAG syntheses and the resulting polymorph of the NIF—CAF cocrystal solvent screening grinding.

Table S2. Solvents used for single crystal growth by solvent evaporation method.

Table S3. Solvents used for the competitive slurry experiments using an equimolar mixture of both polymorphs.

Table S4. Hydrogen bonds for NIF—CAF cocrystal polymorphs [Å and deg.].

Table S5. π,π-stacking interactions analysis of compound NIF—CAF Form II.

Table S6. Maximum apparent solubility (S_{max}) of NIF and its NIF-CAF Cocrystal polymorphs in pH 7.4 phosphate buffer medium.

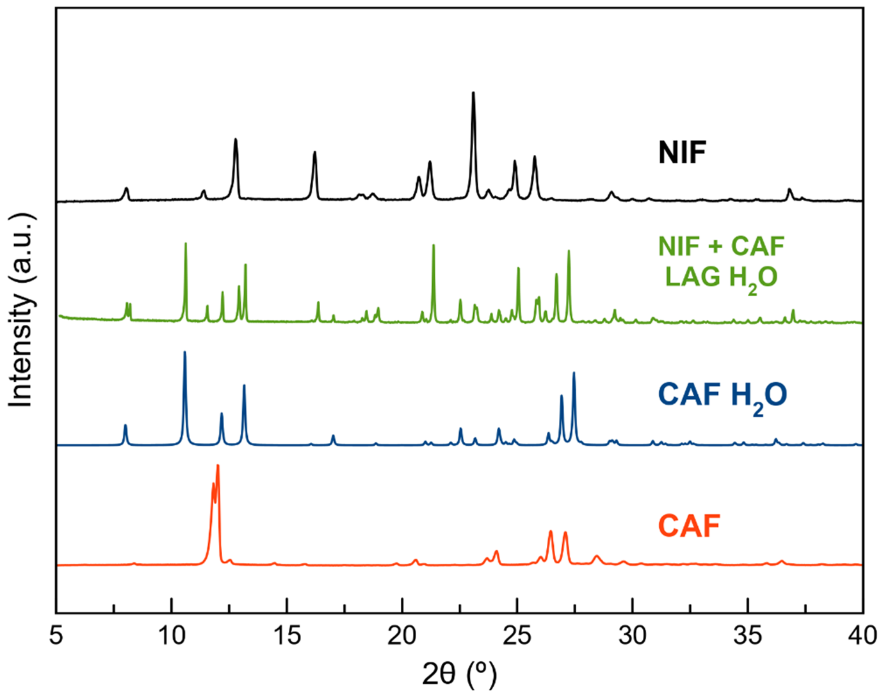

Figure S1. PXRD pattern of the solid material obtained by liquid assisted grinding (LAG) with water, the simulated patterns from crystal structures and the corresponding reactants.

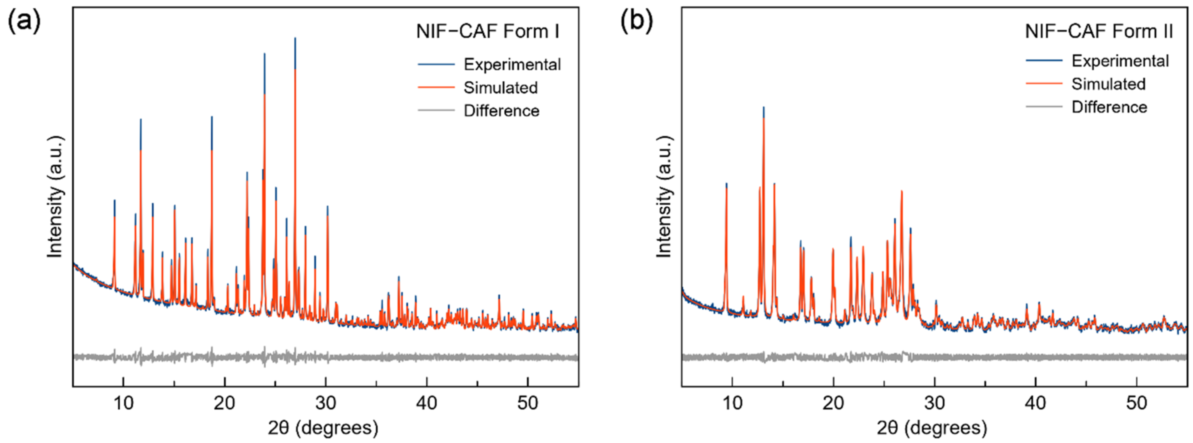

Figure S2. Rietveld profile fit (red line) to the experimental PXRD data (blue line) of NIF–CAF Form I (a) and NIF–CAF Form II (b). The profile fitting for both the cocrystals shows low discrepancy (grey line).

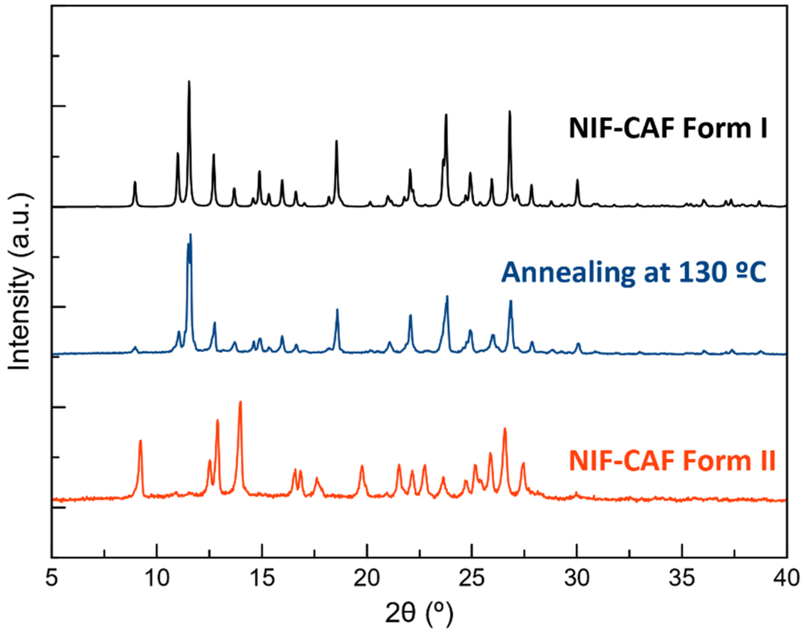

Figure S3. PXRD patterns of NIF–CAF after annealing of Form II at 130 ºC during 24 h.

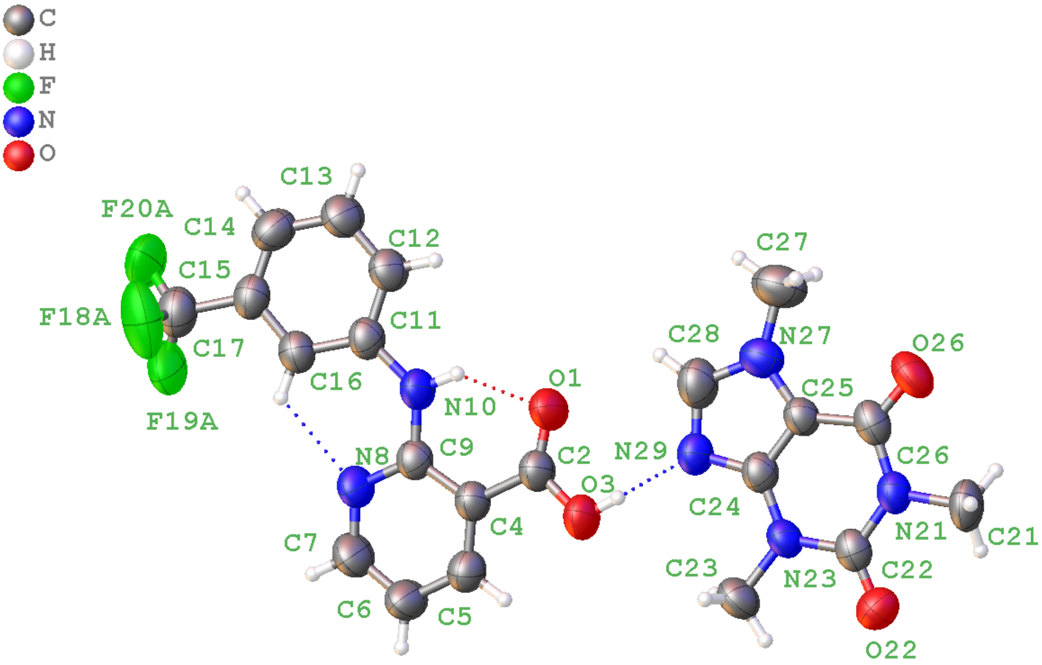

Figure S4. ORTEP representation showing the asymmetric unit of NIF–CAF Form I with atom numbering scheme (thermal ellipsoids are plotted with the 50% probability level, one disordered -CF_3 position omitted for clarity).

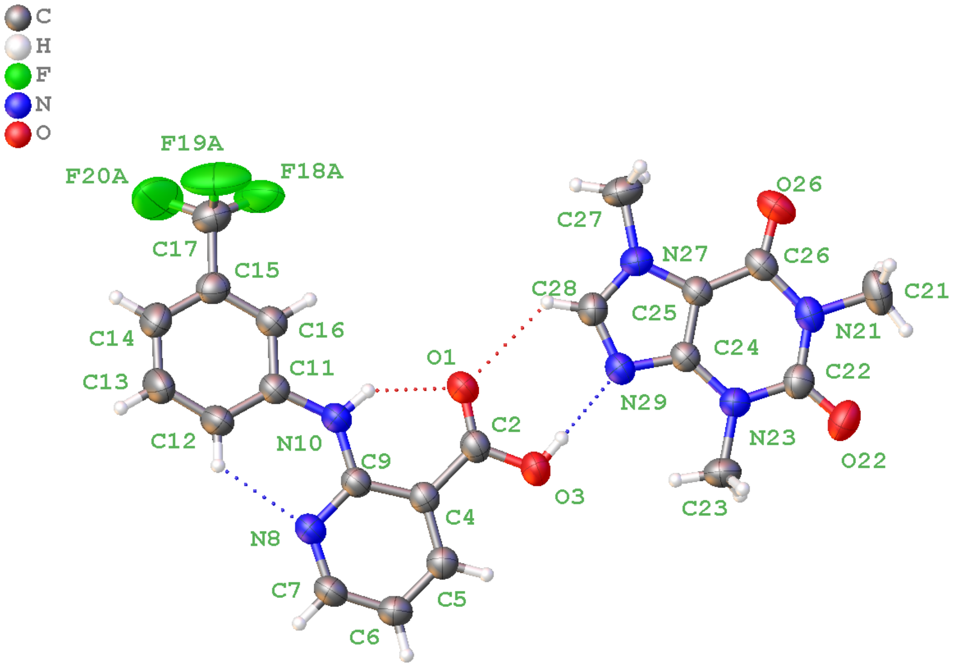

Figure S5. ORTEP representation showing the asymmetric unit of NIF–CAF Form II with atom numbering scheme (thermal ellipsoids are plotted with the 50% probability level, one disordered -CF_3 position omitted for clarity).

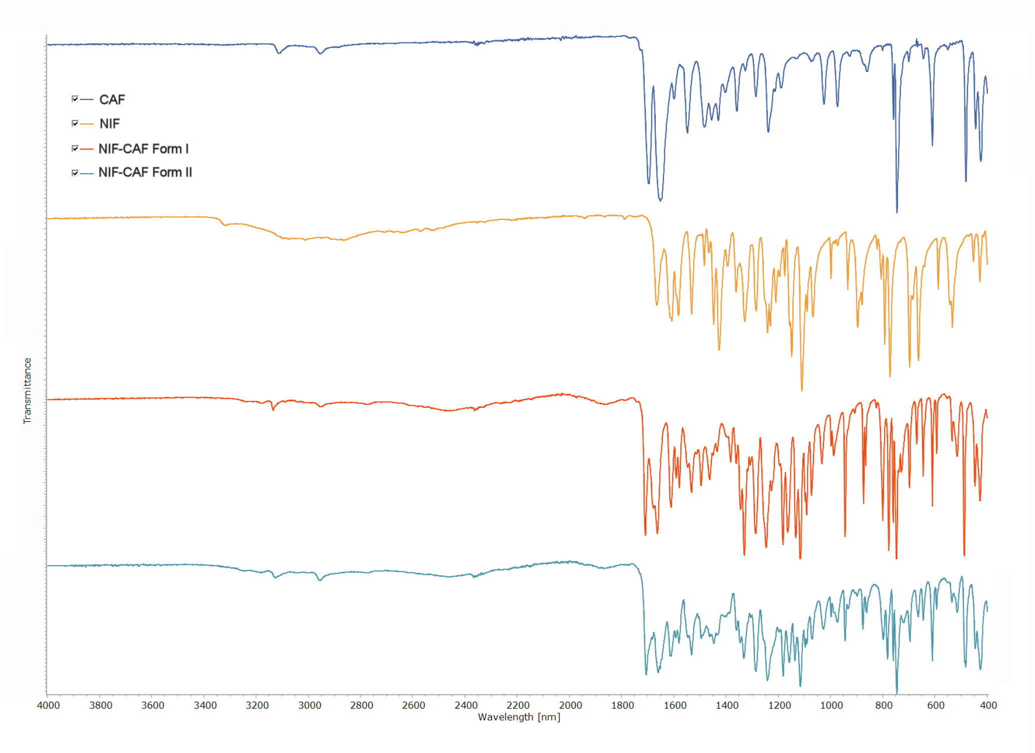

Figure S6. Comparison of Fourier transform infrared (FT−IR) spectra of NIF, CAF and NIF–CAF polymorphs.

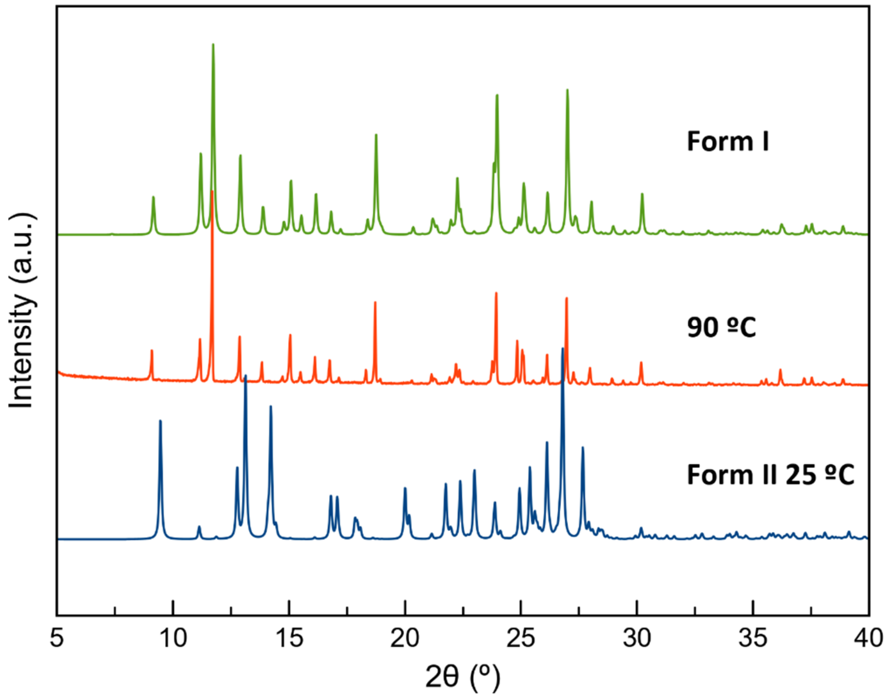

Figure S7. PXRD patterns of the solid material obtained after slurry assay of Form II in octane (OCT) at selected temperature.

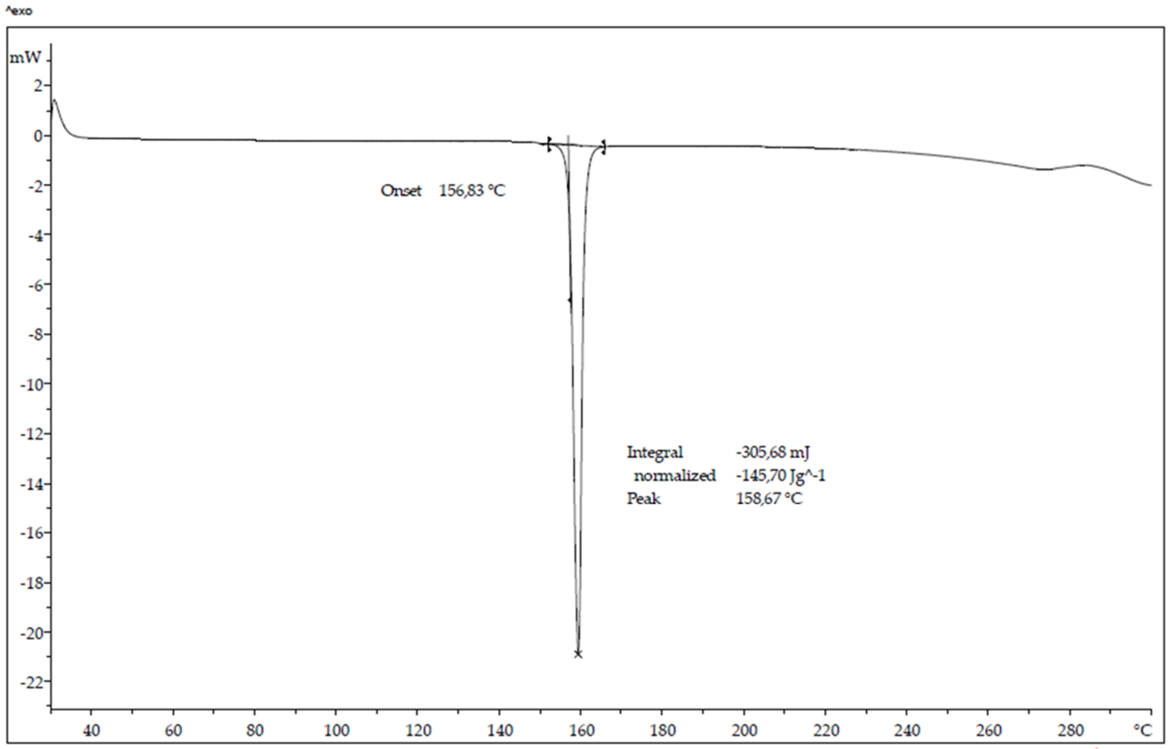

Figure S8. DSC of NIF–CAF Cocrystal Form I.

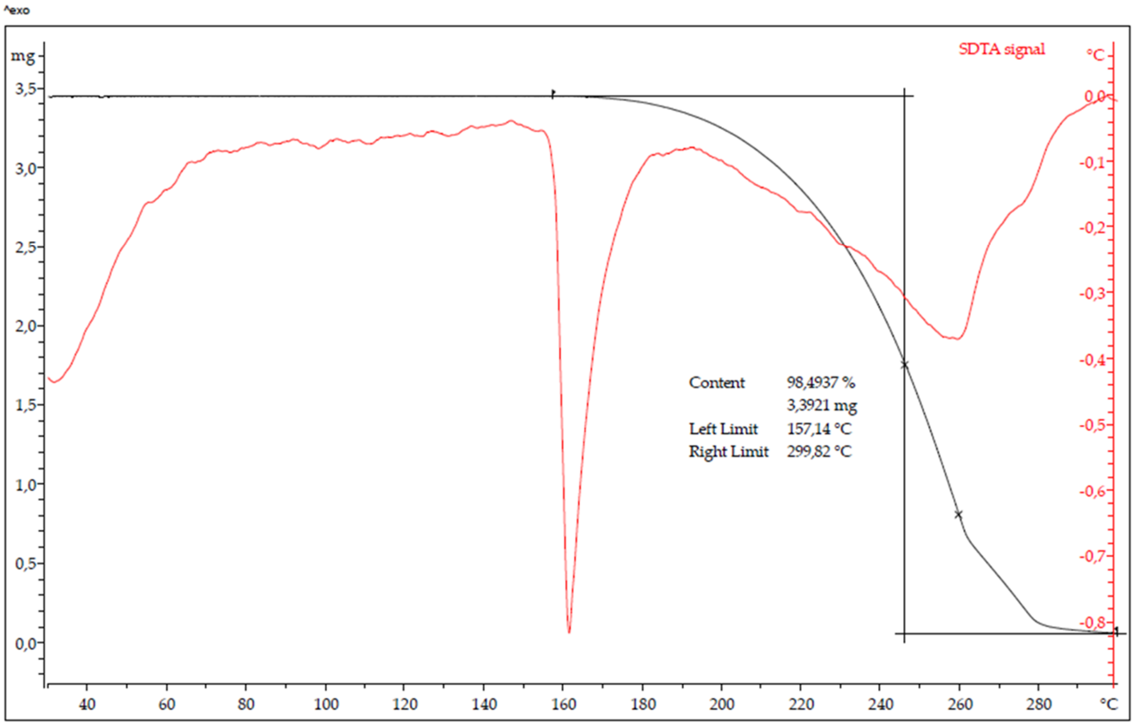

Figure S9. TGA of NIF–CAF Cocrystal Form I.

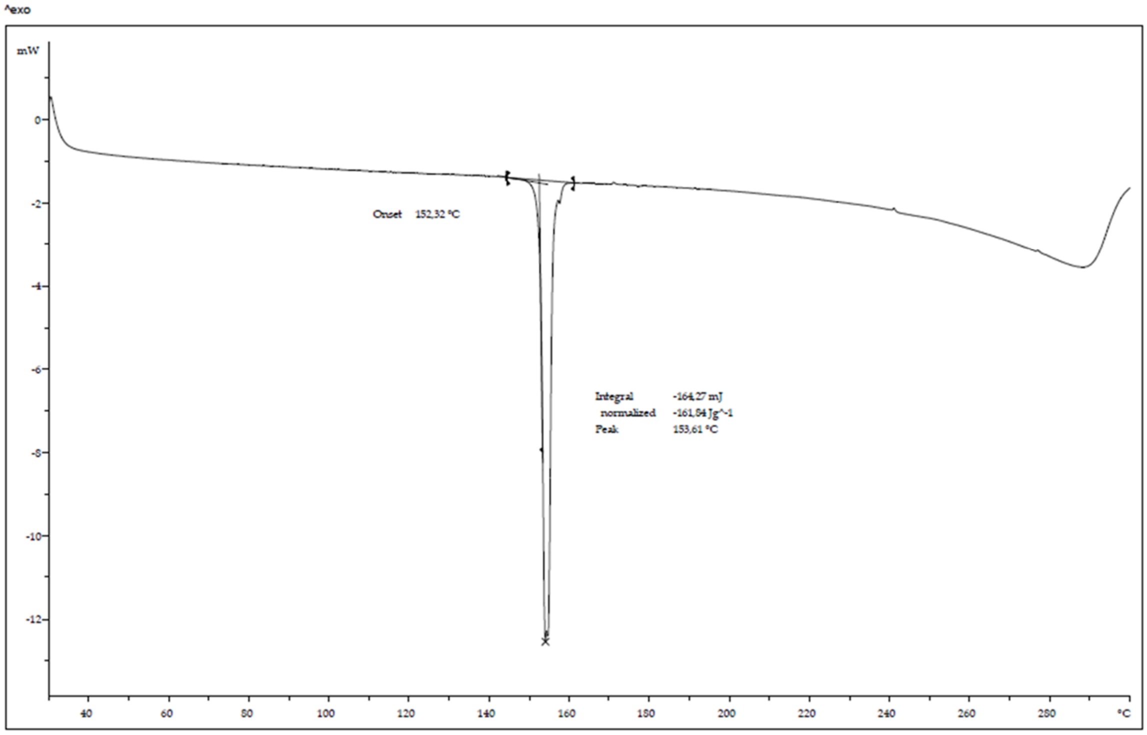

Figure S10. DSC of NIF–CAF Cocrystal Form II.

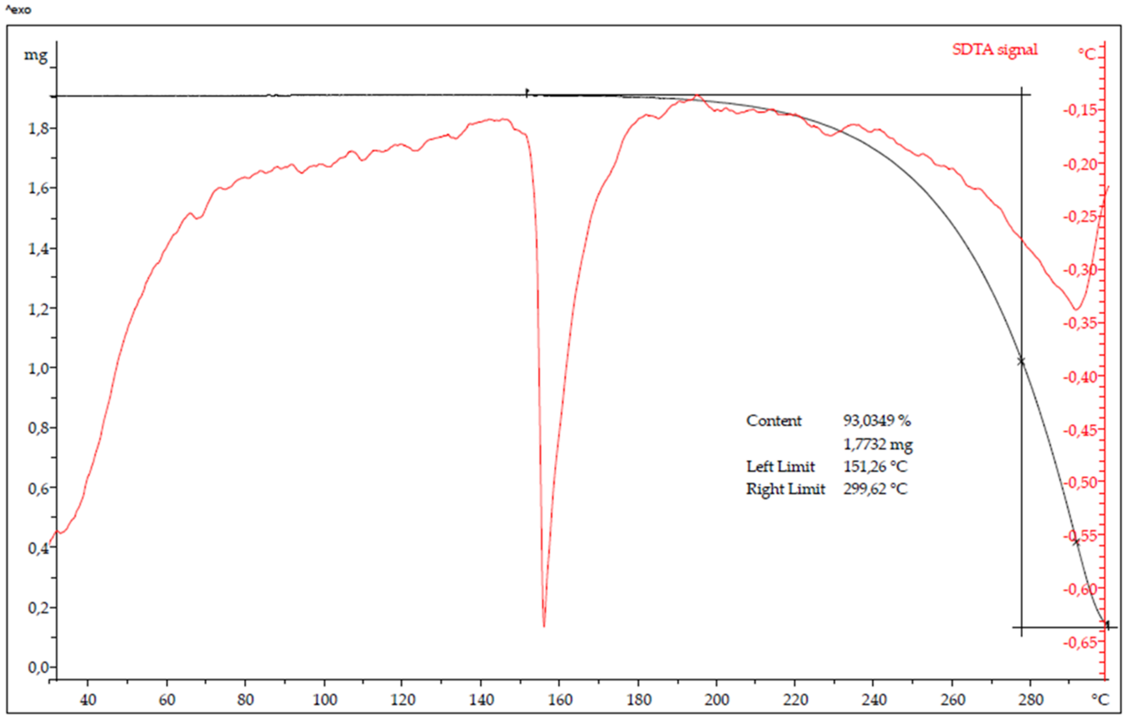

Figure S11. TGA of NIF–CAF Cocrystal Form II.

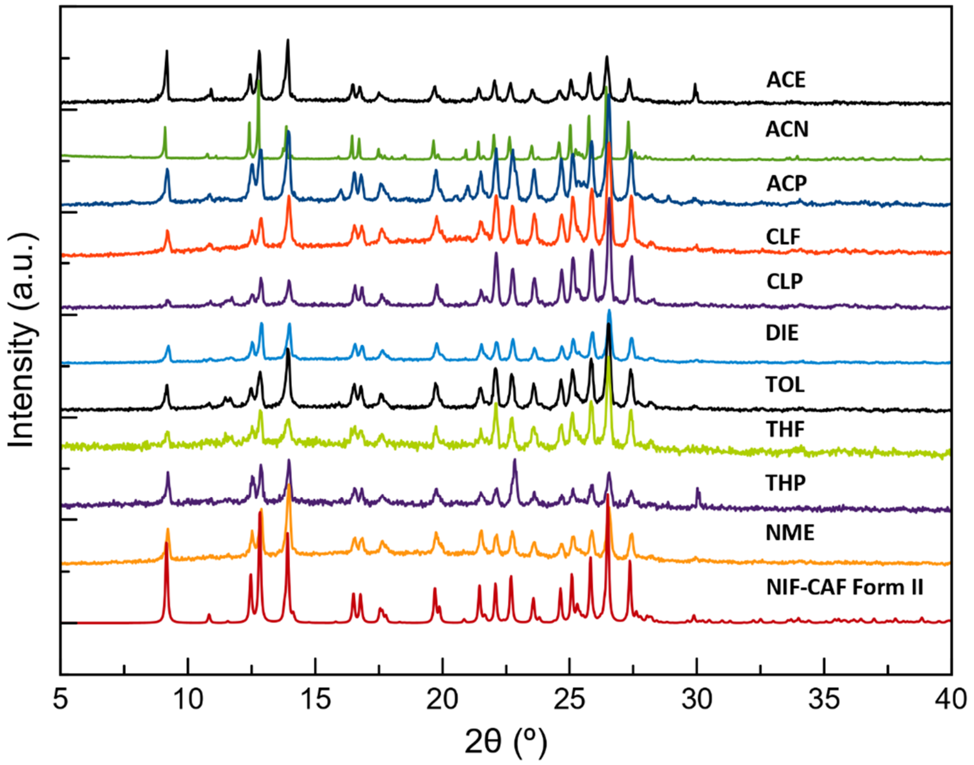

Figure S12. PXRD patterns of NIF–CAF cocrystal forms after the competitive slurry experiments using an equimolar mixture of both polymorphs in a selection of solvents. ACE: acetone; ACN: acetonitrile; ACP: acetophenone; CLF: chloroform; CLP: cyclopentane; DIE: diisopropyl ether; TOL: toluene; THF: tetrahydrofuran; THP: tetrahydropiran; NME: nitromethane.

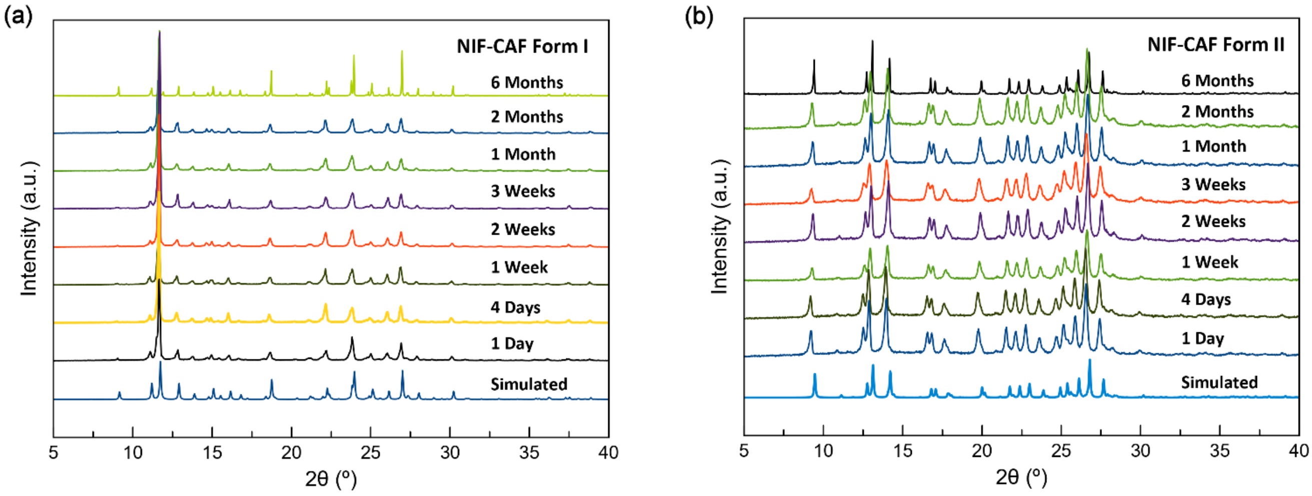

Figure S13. PXRD patterns of NIF–CAF cocrystal polymorphs with respect to the stability under accelerated ageing conditions (40 °C, 75% RH) at different time intervals.

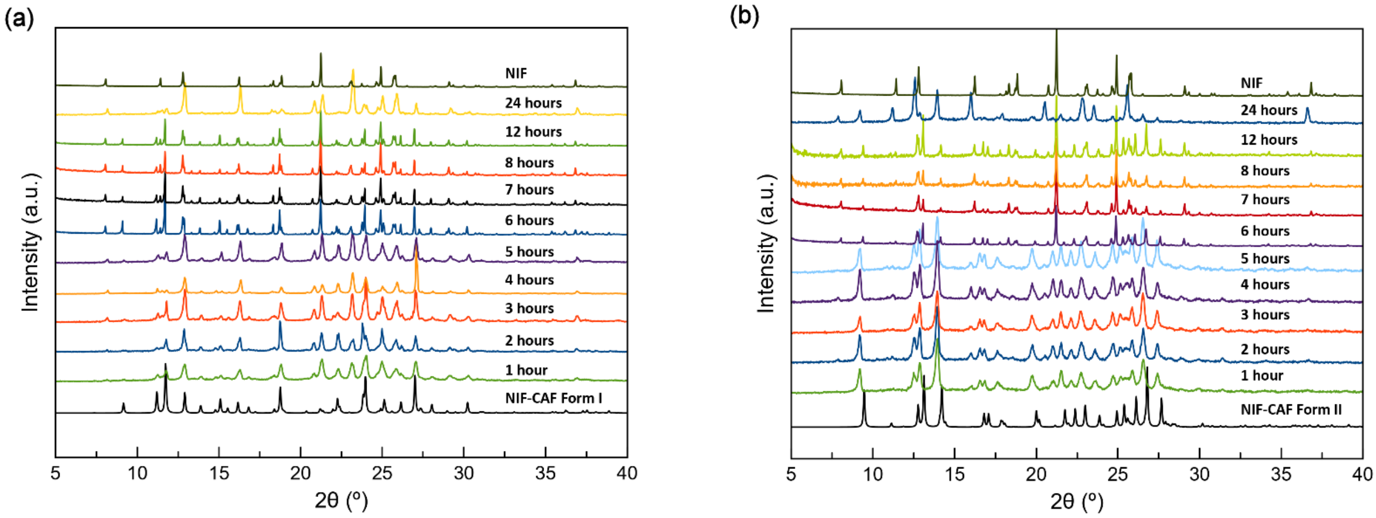

Figure S14. PXRD patterns of NIF–CAF cocrystal polymorphs after the powder dissolution profile assay.

Table S1. Solvents added in the LAG syntheses and the resulting polymorph of the NIF–CAF cocrystal solvent screening grinding.

SOLVENT	NIF–CAF cocrystal polymorph
ACE	Form II
ACN	Form II
ACP	Form I
BNA	Form II
BUT	Form II
CLF	Form I
DCM	Form I
DIE	Form II
EOH	Form II
ETA	Form II
H2O	Mixture of NIF and CAF·H_2O
MET	Form II
NME	Form I
THF	Form II
THP	Form II
TOL	Form II

ACE: acetone; ACN: acetonitrile; ACP: acetophenone; BNA: butanone; CLF: chloroform; DCM: dichloromethane; DIE: diisopropyl ether; EOH: ethanol; ETA; ethylacetate; H2O: water; MET: methanol; NME: nitromethane; THF: tetrahydrofuran; THP: tetrahydropyran; TOL: toluene.

Table S2. Solvents used for single crystal growth by solvent evaporation method.

SOLVENT	Polymorphic outcome	Evaporation rate
ACE	Form I + NIF	Fast
ACN	Form I +NIF	Fast
ACP	Form I + NIF	Fast
DIE	Form II +NIF	Fast
NME	Form I + NIF	Fast
THF	Form I + NIF	Fast
THP	Form I + NIF	Fast
ACN	Form II	Slow

ACE: acetone; ACN: acetonitrile; ACP: acetophenone; DIE: diisopropyl ether; NME: nitromethane; THF: tetrahydrofuran; THP: tetrahydropyran.

Table S3. Solvents used for the competitive slurry experiments using an equimolar mixture of both polymorphs.

SOLVENT	Polymorphic outcome
ACE	Form II
ACN	Form II
ACP	Form I
CLF	Form I
DIE	Form II
NME	Form I
THF	Form II
THP	Form II
TOL	Form II

Table S4. Hydrogen bonds for NIF—CAF cocrystal polymorphs [Å and deg.].

Form I

D-H...A	d(D-H)	d(H...A)	d(D...A)	<(DHA)
O(3)-H(3)···N(29)	0.82	1.86	2.673(4)	173.7
N(10)-H(10)···O(1)	0.86	1.97	2.678(4)	139.2
C(5)-H(5)···O(22)#1	0.93	2.49	3.163(5)	129.3
C(16)-H(16)···N(8)	0.93	2.25	2.843(5)	120.7

Symmetry transformations used to generate equivalent atoms: #1 -x,-y+1,-z

Form II

D-H...A	d(D-H)	d(H...A)	d(D...A)	<(DHA)
O(3)-H(3)···N(29)	0.82	1.87	2.6853(19)	179.9
N(10)-H(10)···O(1)	0.86	1.94	2.663(2)	140.8
C(5)-H(5)···O(003)#1	0.93	2.50	3.171(2)	129.2
C(12)-H(12)···N(8)	0.93	2.34	2.925(3)	120.9
C(28)-H(28)···O(1)	0.93	2.46	3.097(2)	125.8

Scheme 1. -x+1,-y,-z+1 .

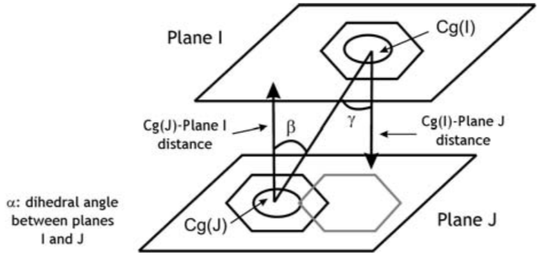

Table S5. a). Analysis of π,π-stacking and C-H··· π interactions analysis of the NIF—CAF cocrystal polymorphs.

Form I

6-Membered Ring (2) C11 --> C12 --> C13 --> C14 --> C15 --> C16 -->
5-Membered Ring (3) N27 --> C25 --> C24 --> N29 --> C28 -->
6-Membered Ring (4) N21 --> C22 --> N23 --> C24 --> C25 --> C26 -->

Analysis of Short Ring-Interactions with Cg-Cg Distances < 6.0 Ang., Alpha < 20.000 Deg. and Beta < 60.0 Deg.

Cg(I)	Res(I)	Cg(J)	[ARU(J)]	Cg-Cg	Transformed J-Plane P, Q, R, S	Alpha	Beta	Gamma	CgI_Perp	CgJ_Perp	Slippage
Cg2	[1] ->	Cg3	[1556.02]	3.661(3)	0.2689 0.8551 0.4432 13.5770	5.0(2)	14.5	19.4	3.4538(16)	3.5434(16)	0.920
Cg2	[1] ->	Cg4	[1556.02]	3.730(3)	0.2692 0.8440 0.4640 13.6863	4.31(18)	22.2	22.3	3.4504(16)	3.4525(15)	1.411
Cg3	[2] ->	Cg2	[1554.01]	3.661(3)	0.2023 0.8430 0.4984 5.7651	5.0(2)	19.4	14.5	3.5434(16)	3.4537(16)	1.214
Cg4	[2] ->	Cg2	[1554.01]	3.730(3)	0.2023 0.8430 0.4984 5.7651	4.31(18)	22.3	22.2	3.4526(15)	3.4503(16)	1.416
Cg4	[2] ->	Cg4	[3665.02]	3.515(2)	-0.2692-0.8440-0.4640 -12.9569	0.00(17)	13.6	13.6	3.4164(15)	3.4164(15)	0.825

[1556] = X,Y,1+Z
[1554] = X,Y,-1+Z
[3665] = 1-X,1-Y,-Z

Analysis of Y-X...Cg(Pi-Ring) Interactions (X..Cg < 4.0 Ang. - Gamma < 30.0 Deg)

Y--X(I)		Res(I)	Cg(J)	[ARU(J)]	X..Cg	Transformed J-Plane P, Q, R, S	X-Perp	Gamma	Y-X..Cg	Y..Cg	Y-X,Pi
C22	-O22	[2] ->	Cg1	[1554.01]	3.425(4)	0.3668 0.8345 0.4112 6.6051	3.284	16.46	105.6(3)	3.931(5)	0.42
C22	-O22	[2] ->	Cg3	[3665.02]	3.407(4)	-0.2689-0.8551-0.4432 -13.1133	3.354	10.09	84.4(2)	3.504(5)	2.49
				Min or Max	3.407		3.284	10.1	105.60	3.504	2.49

[1554] = X,Y,-1+Z
[3665] = 1-X,1-Y,-Z

Table S5(b). Analysis of π,π-stacking and C-H··· π interactions analysis of the NIF—CAF cocrystal polymorphs.

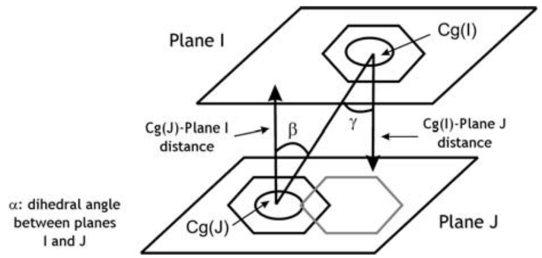

Form II

6-Membered Ring (1)	N8	-->	C7	-->	C6	-->	C5	-->	C4	-->	C9	-->
6-Membered Ring (2)	C11	-->	C12	-->	C13	-->	C14	-->	C15	-->	C16	-->
5-Membered Ring (3)	N27	-->	C25	-->	C24	-->	N29	-->	C28	-->		
6-Membered Ring (4)	N21	-->	C22	-->	N23	-->	C24	-->	C25	-->	C26	-->

==

Analysis of Short Ring-Interactions with Cg-Cg Distances < 6.0 Ang., Alpha < 20.000 Deg. and Beta < 60.0 Deg.

==

Cg(I)	Res(I)	Cg(J)	[ARU(J)]	Cg-Cg	Transformed J-Plane P, Q, R, S	Alpha	Beta	Gamma	CgI_Perp	CgJ_Perp	Slippage
Cg1	[1] ->	Cg3	[1655.02]	3.6704(11)	-0.4606 0.8334 0.3055 1.5905	1.08(10)	23.0	21.9	3.4049(8)	3.3786(7)	1.434
Cg3	[2] ->	Cg1	[1455.01]	3.6705(11)	-0.4731 0.8313 0.2916 7.9751	1.08(10)	21.9	23.0	3.3785(7)	3.4050(8)	1.371
Cg3	[2] ->	Cg2	[1445.01]	4.1995(12)	-0.3238 0.8766 0.3559 1.7909	8.72(10)	32.7	24.9	3.8103(7)	3.5342(8)	2.268
Cg4	[2] ->	Cg1	[1445.01]	4.2087(11)	-0.4731 0.8313 0.2916 1.2227	0.44(8)	36.2	36.5	3.3839(7)	3.3969(8)	2.485
Cg4	[2] ->	Cg1	[1455.01]	4.2702(11)	-0.4731 0.8313 0.2916 7.9751	0.44(8)	37.5	37.9	3.3685(7)	3.3882(8)	2.599

[1655] = 1+X,Y,Z
[1445] = -1+X,-1+Y,Z
[1455] = -1+X,Y,Z

==

Analysis of Y-X...Cg(Pi-Ring) Interactions (X..Cg < 4.0 Ang. - Gamma < 30.0 Deg)

==

Y--X(I)	Res(I)	Cg(J)	[ARU(J)]	X..Cg	Transformed J-Plane P, Q, R, S	X-Perp	Gamma	Y-X..Cg	Y..Cg	Y-X,Pi
C17 -F20A	[1] ->	Cg2	[2755.01]	3.794(10)	0.3238 0.8766-0.3559 8.7483	-3.480	23.48	149.4(7)	4.983(3)	81.46
C22-O22	[2] ->	Cg1	[1445.01]	3.4403(17)	-0.4731 0.8313 0.2916 1.2227	3.419	6.40	86.78(11)	3.5840(19)	0.98
C17-F20B	[1] ->	Cg2	[2755.01]	3.83(2)	0.3238 0.8766-0.3559 8.7483	-3.700	15.03	150.6(11)	4.983(3)	58.91

[2755] = 5/2-X,1/2+Y,1/2-Z
[1445] = -1+X,-1+Y,Z

Table S6. Maximum apparent solubility (S_{max}) of NIF and its NIF-CAF Cocrystal polymorphs in pH 7.4 phosphate buffer medium.

	S_{max} (mg·mL^{-1})	Solubility enhancement
NIF	2.25	
Form I	24.39	10.84X
Form II	21.51	9.56X
